# Proto-Object Based Saliency Model With Texture Detection Channel

**DOI:** 10.3389/fncom.2020.541581

**Published:** 2020-09-24

**Authors:** Takeshi Uejima, Ernst Niebur, Ralph Etienne-Cummings

**Affiliations:** ^1^The Department of Electrical and Computer Engineering, The Johns Hopkins University, Baltimore, MD, United States; ^2^The Solomon Snyder Department of Neuroscience and the Zanvyl Krieger Mind/Brain Institute, The Johns Hopkins University, Baltimore, MD, United States

**Keywords:** saliency, visual attention, image texture analysis, neuromorphic engineering, proto-object

## Abstract

The amount of visual information projected from the retina to the brain exceeds the information processing capacity of the latter. Attention, therefore, functions as a filter to highlight important information at multiple stages of the visual pathway that requires further and more detailed analysis. Among other functions, this determines where to fixate since only the fovea allows for high resolution imaging. Visual saliency modeling, i.e. understanding how the brain selects important information to analyze further and to determine where to fixate next, is an important research topic in computational neuroscience and computer vision. Most existing bottom-up saliency models use low-level features such as intensity and color, while some models employ high-level features, like faces. However, little consideration has been given to mid-level features, such as texture, for visual saliency models. In this paper, we extend a biologically plausible proto-object based saliency model by adding simple texture channels which employ nonlinear operations that mimic the processing performed by primate visual cortex. The extended model shows statistically significant improved performance in predicting human fixations compared to the previous model. Comparing the performance of our model with others on publicly available benchmarking datasets, we find that our biologically plausible model matches the performance of other models, even though those were designed entirely for maximal performance with little regard to biological realism.

## Introduction

Human eyes capture and send large amounts of data from the environment to the brain, more than can be processed in detail. To deal with the overwhelming quantity of input, various stages of visual processing select a small subset of all available information for detailed processing and discard the remainder, for reviews see Desimone and Duncan (1995), Reynolds and Chelazzi (2004), Petersen and Posner (2012). Understanding quantitatively how the brain selects important information, and where humans fixate, is an important research topic in neuroscience and computer vision. In a seminal study, Koch and Ullman (1985) laid the basis for understanding the mechanisms of selective attention in terms of biologically plausible neuronal circuitry which led to the development of detailed computational models of this process (Niebur and Koch, 1996; Itti et al., 1998; Itti and Koch, 2001). A better understanding of visual attentive selection will improve the effectiveness of graphic arts, advertisements, traffic signs, camouflage and many other applications, as well as contributing to the basic science goal of understanding visual processing in the brain.

Because only the fovea allows for high resolution imaging, the observer needs to move the eyeballs for thorough scene inspection. Therefore, eye movements can be used to observe what is attended (i.e., what is interesting and important for the brain). There is a nexus between the modeling of visual saliency and the detection of salient objects. The goal of modeling visual saliency is to understand and predict what attracts human attention and gaze (Parkhurst et al., 2002; Bylinskii et al., 2016). Even though changes of covert attention (without eye movements) and overt attention (with eye movements) are not the same, as has been known since the nineteenth century [see von Helmholtz (1896); the pertinent section was translated by Nakayama and Mackeben (1989)], under natural viewing conditions they are positively correlated. In visual saliency modeling, the locations and objects which humans fixate when visually inspecting an image or scene are therefore considered as a manifestation of what is considered to be salient. Maps generated from the fixations of human observers are then considered to be ground truth for which parts of the scene or image are attended (Parkhurst et al., 2002). In computer vision, salient object detection aims to find the most salient object in images (Liu et al., 2011; Borji et al., 2019). In this case, ground truth is usually a pixel-wise or box-shaped binary mask which indicates the most attended object or place. The focus of this paper is on the first of these problems (i.e., to model the mechanisms that govern the deployment of attention by humans). Our measure of model performance will be its performance in predicting human eye movements, the aforementioned ground truth measure. A basic idea is that an ideal model would be able to predict fixations as well as other subjects (Wilming et al., 2011). Importantly, our main objective is to gain understanding of the mechanisms underlying attentional selection in human (biological) information processing, rather than optimizing prediction performance on benchmark data sets.

One limitation of our approach is that our model includes only bottom-up processes, even though visual attention is guided by both bottom-up and top-down information flow. The bottom-up process is a data-driven, non-intentional, and reflexive process, based on low-level features (Koch and Ullman, 1985). The top-down process is task dependent and controlled by an organism's internal state and its goals (Yarbus, 1967). Substantial efforts have been made to unify both processes (Navalpakkam and Itti, 2005, 2006; Chikkerur et al., 2010; Kollmorgen et al., 2010) but top-down influences are much harder to control and to measure experimentally. Our model is therefore concerned only with bottom-up attention.

Various methods have been proposed to predict local saliency, such as graph based models (Harel et al., 2007) and high-level feature based models (Judd et al., 2009). Deep neural networks (DNNs) for visual saliency computation have also been studied in recent years, and they show promising results predicting human attention (Kümmerer et al., 2014, 2016; Vig et al., 2014; Huang et al., 2015; Kruthiventi et al., 2015; Cornia et al., 2016). However, it is difficult to understand how DNN-based algorithms process the visual information, and the structure of the algorithm is to a large extent opaque to the researcher. The possible extent of its role in providing understanding how the brain performs the same task is therefore not clear.

In-between these two levels of abstraction—high-level fully-developed complex structures and simple local feature contrast—there lies a highly promising approach to understanding human perception, commonly referred to as Gestalt perception. Nearly a century ago psychologists formulated “Gestalt laws” which determined, among other things, where humans direct attention. There is no universal set of laws, but examples of salient objects are those possessing qualities like good continuation, proximity, closure, symmetry etc. (Wertheimer, 1923; Koffka, 1935). Russell et al. incorporated Gestalt principles into visual saliency modeling (Russell et al., 2014). In their model, proximity, continuity, and convexity are implicitly used in a grouping mechanism that segregates foreground (proto-)objects from the background and also predicts human attentional selection well (Russell et al., 2014) (see next section for the distinction between objects and proto-objects).

The Russell et al. model originally made use only of intensity, color, and orientation modalities (see below for later additions). In this report, we extend it by adding channels which incorporate nonlinear texture features. Since modeling of texture is quite complex (Simoncelli and Portilla, 1998), we have developed a compact representation that can easily be computed in real-time or realized in hardware.

## Related Work

### Biologically-Plausible Saliency Model

One of the early and influential saliency models (Itti et al., 1998) relies on the low-level features of intensity, color, and orientation. The model uses center-surround cells and normalization operators to determine conspicuous locations for each feature space. The final saliency map is obtained by normalizing and summing results of the contributions from all feature maps.

Going beyond that approach, it was recognized that visual scenes are commonly thought to be organized in terms of objects. Humans frequently fixate at centers of objects rather than their edges even though the latter generally have higher contrast than their interiors (Einhäuser et al., 2008; Nuthmann and Henderson, 2010; Stoll et al., 2015), see however Borji et al. (2013a). This would imply that humans do not simply fixate high contrast areas but at objects. It was argued by Rensink (2000) and by Zhou et al. (2000) that perceptual organization does not require the formation of fully-formed objects; those would be needed only for tasks like object recognition, semantic categorization or discrimination. Instead, to organize a visual scene, it is sufficient to segment it into entities that are characterized by a few elementary features, like their position, size etc. Following Rensink (2000), we call these entities “proto-objects.” Since we will never make reference to objects (as opposed to proto-objects) in this study, we will use the terms “object” and “proto-object” interchangeably for the sake of simplicity.

We should note here that the meaning of “proto-objects” in this paper is different from the one in Walther and Koch (2006). In the current paper, and in the Russell et al. model, discussed in the next section, a saliency map is computed from proto-objects which are obtained from feature maps. In contrast, the Walther and Koch model computes proto-objects from a saliency map. Thus, the flow of information between saliency maps and proto-objects is opposite.

One recent model of attentional selection is based on the Gestalt law of “surroundedness,” making use of the fact that surrounded regions are more likely to be seen as figure (foreground) than non-surrounded regions, and more likely to be attended. The resultant Boolean Map based Saliency model (BMS) compared favorably with other attentional models (Zhang and Sclaroff, 2016). Craft et al. used a more general approach to employ Gestalt principles (Craft et al., 2004). To implement convexity and proximity, border-ownership selective cells and grouping cells were introduced, resulting in foreground objects (figures) having higher saliency values than the background. Russell et al. (2014) built on this model to generate an image-computable model which uses intensity, color, and orientation channels. This latter model has been extended by addition of a motion channel (Molin et al., 2013) as well as a depth channel (Hu et al., 2016; Mancinelli et al., 2018).

The use of (proto-)objects for fixation prediction can be justified from a different viewpoint. DeepGaze2, one of the most successful DNN-based saliency models, uses the VGG-19 network which is pre-trained for object recognition. Its fully-connected layers are removed, and a readout network is added at the top of the convolution layers and trained with the SALICON and MIT1003 networks to predict human fixations (Kümmerer et al., 2016). That is, the feature extraction layer of DeepGaze2 is the same as that of an object recognition network, while the “decoding” layers are different. Even though it is difficult to understand what is represented in detail in the convolution layers, it is safe to say that these features are suitable for object recognition given that the VGG-19 shows good performance in the object categorization task. DeepGaze2 shows very high performance in fixation prediction even though it leaves the VGG-19 convolution layers unchanged, and is only trained in the added readout layers. This means that the features appropriate for object recognition are also useful for fixation prediction. This is further evidence that there is a strong relation between objects and attention.

**Figure 1:**
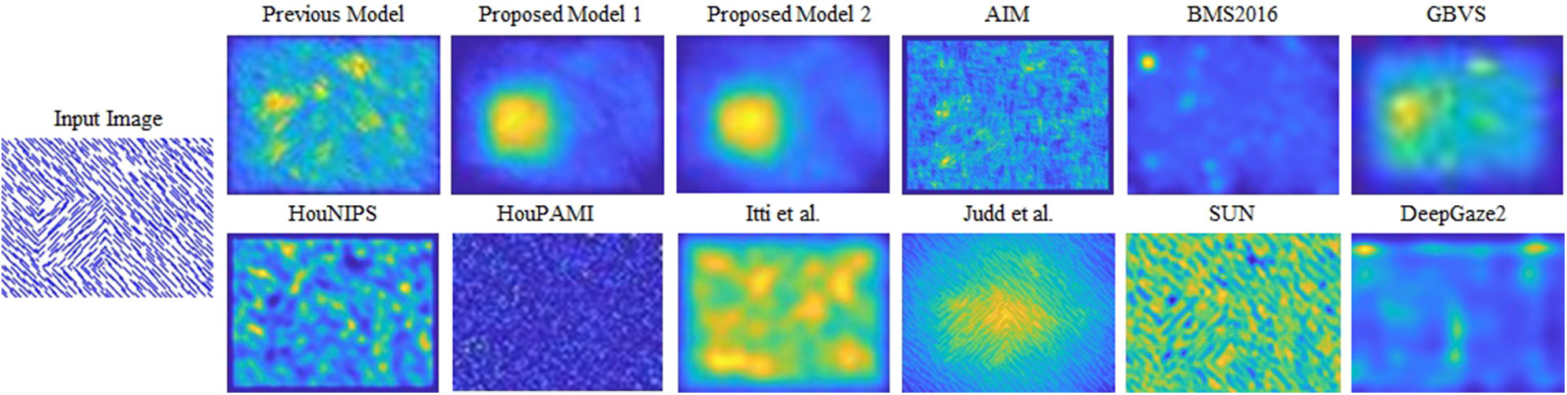
Saliency maps of the models compared in this study for a synthesized input image. The pattern-defined figure (a square) is in the lower-left of the input image. Neurophysiological data show that primate V1 neuron activity is enhanced when the receptive fields are within the figure. Different from previous proto-object model and other saliency models, our model can discriminate the pattern-defined figure from the background. The cited models: AIM (Bruce and Tsotsos, 2005), BMS2016 (Zhang and Sclaroff, 2016), GBVS (Harel et al., 2007), HouNIPS (Hou and Zhang, 2008), HouPAMI (Hou et al., 2012), Itti et al. originally from Itti et al. (1998) but we used the latest code from Harel et al. (2007), Judd et al. (2009), SUN (Zhang et al., 2008), and DeepGaze2 (Kümmerer et al., 2016).

Proto-object based models, however, fail to capture second-order features such as a pattern-defined object as shown in Figure 1. Because the average luminance of the foreground figure is identical to that of the background at relevant spatial scales, simple center-surround differences cannot detect such pattern-defined objects. In the example shown in Figure 1, orientation-selective cells which are modeled as Gabor filters can detect the oriented lines but not the square defined by the lines. Therefore, another layer with larger receptive fields (RF) is needed to capture high-order features.

On the other hand, monkey V1 cells can discriminate the pattern-defined foreground from the background (Lamme, 1995). In Lamme's study, the V1 neuron response is enhanced when its RF is on the boundary or within the figure defined by the oriented lines, compared to the response when the RF is on the background even though the figure is much larger than the RF of the neuron. This enhancement occurs later (30–40 ms) than the initial response to the stimuli. The enhancement for the surface of the figure vanishes when V3 or higher cortical areas are lesioned, but the response for the boundary of the figure does not (Lamme et al., 1998). This suggests that the enhancement for the surface of the figure is a consequence of feedback from V3 or higher cortical areas, while enhancement of the figure border is caused by feedback from V2 or through recurrent interaction within V1 (Lee and Yuille, 2006). Such perception of a (proto-) object defined by pattern will attract attention. In the human brain, bottom-up orienting is processed in the ventral attention system which includes the temporo-parietal junction and ventral frontal cortex (Corbetta and Shulman, 2002; Petersen and Posner, 2012; Shomstein, 2012). This pathway projects to the frontal eye field and controls movement of the eyeballs, in interaction with top-down attention.

### Texture Characterization

Describing texture mathematically is not trivial and algorithmically complex. For example, Julesz proposed n-th-order statistics methods to describe texture features which can be distinguishable or indistinguishable for humans (Julesz, 1975). Wavelet analysis and statistical methods are also used (Simoncelli and Portilla, 1998; Van De Wouwer et al., 1999; Portilla and Simoncelli, 2000). More recently, sparse representation concepts have been used to tackle the representation of texture (Schaeffer and Osher, 2013; Ono et al., 2014; Zhang and Patel, 2018). In all these cases, the models are complex and not easily reduced to a simple construct that can be easily folded into a model of human visual attention, especially if that model is supposed to be biologically plausible. In this paper, we propose simplified descriptions of texture that can be incorporated into cortical models of visual attention, particularly our previous proto-object based visual saliency model (Russell et al., 2014).

How are the mechanisms that represent texture implemented in biological vision? Bergen and Adelson have suggested that human texture perception may be explained by relatively simple center-surround cells of different scales (Bergen and Adelson, 1988). Also, the nervous system may process second-order textures by rectification and second-stage orientation-selective filters following first-stage filters (Sutter et al., 1995; Mareschal and Baker, 1998). This concept can be thought of as the combination of simple cells (S-cells) and complex cells (C-cells) (Hubel and Wiesel, 1962). A similar mechanism is also used in the HMAX algorithm proposed as a biologically realistic model for object recognition (Riesenhuber and Poggio, 1999). It uses a cascade of orientation-selective S-cells with small RFs and max operation C-cells which have larger RFs. Similarly, the combination of a rectification linear unit (ReLU) and max pooling has become common in deep neural networks (Krizhevsky et al., 2012). Although the biological plausibility of max pooling is still controversial, efforts to construct biologically plausible models based on neural networks have been reported (Yu et al., 2002), and neurophysiological results support the presence of max pooling mechanisms in visual cortex (Pestilli et al., 2011). The importance of spatial pooling for texture segregation is also suggested in other studies (Bergen and Landy, 1991). Furthermore, recent experiments have revealed that human and macaque V2 has an important role in discriminating complicated texture, and the perception of texture depends on the energy of the product of orientation-selective filters (Freeman et al., 2013). Our present model adopts these concepts because they are simple and suitable for integration into the proto-object based model (Russell et al., 2014). Furthermore, they can be realized in hardware or highly efficient software implementations.

## Model

As mentioned above, our model is based on the Russell et al. model, and the proto-object mechanism is the same. Here, we focus on texture feature extraction and the relation between the algorithm and biological systems. See Supplementary Materials and the original paper (Russell et al., 2014) for details of the basic algorithm.

### Intensity and Color Feature Extraction

We are concerned with photopic vision, the light intensity range above ~3 cd/m^2^ in which rod photoreceptors are saturated, and therefore play no role, and all information is provided by cones. Color and intensity information originates in three types of cones which are sensitive to long- (L), middle-(M), and short- (S) wavelengths, respectively. After intra-retinal processing, retinal ganglion cells convey all information to the brain. The largest projection is *via* the optic nerve to the thalamus, specifically the lateral geniculate nucleus (LGN) which, in turn, projects via the optic radiation to the primary visual cortex, area V1.

The retinal ganglion cells mostly consist of three types: parasol, midget, and bistratified (Nassi and Callaway, 2009). The parasol cells are the beginning of the magno-cellular pathway in the LGN and receive input from L and M cones. They represent mainly intensity (luminance) information and are highly sensitive to motion signals. The midget and bistratified cells project to parvo- and konio-cellular populations of the LGN and transfer shape as well as chromatic information, the latter in red-green and yellow-blue pathways. This representation is thought to reduce redundancy caused by sensitivity profiles of cones and to represent natural scene spectral components efficiently (Webster and Mollon, 1997; Lee et al., 2002). We model this mechanism by computing one intensity channel and four color opponency channels, red-green (RG), green-red (GR), blue-yellow (BY), and yellow-blue (YB).

We model the intensity channel, J, as:

(1) $$\begin{array}{l} J=\frac{r+g+b}{3} \end{array}$$

where *r*, *g*, and *b* are the red, green, and blue components of the RGB input image, in a coarse analogy to the L, M, and S cones (Itti et al., 1998).

Since hue variations are not perceivable at very low luminance, a color signal is only computed for pixels whose intensity value is greater than 10% of the global intensity maximum of the image. The four color opponency channels, RG, GR, BY, and YB, are obtained from the tuned color channels; red (R), green (G), blue (B), yellow (Y) as follows:

(2) $$\begin{array}{l} R=\left\lfloor r-\frac{g+b}{2}\right\rfloor ,\;G=\left\lfloor g-\frac{r+b}{2}\right\rfloor \\ B=\left\lfloor b-\frac{r+g}{2}\right\rfloor ,\;Y=\left\lfloor \frac{r+g}{2}-\frac{\left|r-g\right|}{2}-b\right\rfloor \end{array}$$

(3) $$\begin{array}{l} \mathrm{RG}=\left\lfloor R-G\right\rfloor ,\;\mathrm{GR}=\left\lfloor G-R\right\rfloor \\ BY=\left\lfloor B-Y\right\rfloor ,\;\mathrm{YB}=\left\lfloor Y-B\right\rfloor \end{array}$$

where [·] is half-wave rectification.

For the intensity feature, the red, green, and blue components in RGB images do not correspond directly to the three described types of cones, and luminance perception is not a simple average of responses of three types of cones. Likewise, for the color feature, Equations (2), (3) are a simplified version of biological color opponency computation. To use other color conversion schemes (e.g., the CIE Lab color space) is a possible alternative. We use these definitions for the sake of simplicity and also because they are the same as those used by Itti et al. (1998), Russell et al. (2014), which makes it easy to compare the proposed model with those previous studies.

We do not implement temporal dynamics of neuronal activity because all our stimuli are static images. Other saliency models have been proposed that do include image motion, for instance, Molin et al. (2015).

Since salient objects can appear at different scales, the algorithm successively down-samples the intensity and color opponency channels in half-octaves (steps of $\sqrt{2}$) to form an image pyramid spanning three octaves. We represent these pyramid images as $J^{k}$, $RG^{k}$, $GR^{k}$, $BY^{k}$, and $YB^{k}$ where *k* is the level of the pyramid.

### Texture Channels for the Proto-Object Based Saliency Model

The model we propose in this paper includes texture channels which are based on visual processing mechanisms in cortical areas V1 and V2. Thalamic magno-cellular neurons project to layer 4Cα of the V1 cortex, and parvo-cellular and konio-cellular neurons to layer 4Cβ and also directly to 2/3. Here and in the next layer (layers 4B and 2/3), many cells are orientation selective. We first discuss simple cells that are modeled as Gabor filters (Kulikowski et al., 1982), other cell types are discussed below. Even-symmetric Gabor filters are used to build intensity and color opponency pyramids $J^{k}$, $RG^{k}$, $GR^{k}$, $BY^{k}$, and $YB^{k}$ as described above. Odd Gabor filters are suitable to detect an object's boundary such as luminance change, and the even-symmetric Gabor filters are sensitive to lines. Texture features exist inside an object, and even-symmetric Gabor filters are suitable to capture such stimuli.

Whether the early stages of the human brain use the features of the combination of color and orientation is unclear. Treisman's famous experiments which led her to develop feature integration theory suggests that the brain needs attention to bind color and orientation features, and does not use the combination of them as basic features in early visual processing (Treisman and Gelade, 1980). We, however, found that including the texture channels derived from color opponency improves the performance of the model. We will revisit this argument in the Discussion section.

The Gabor filtered intensity and color opponency maps are represented as:

(4) $$\begin{array}{l} F_{\theta ,\;C}^{k}\left(x,y\right)=|C^{k}\left(x,y\right)\;*\;g_{\;e,\;\theta }\left(x,y\right)|\\ \left(C=\left\{J,\mathrm{RG},\mathrm{GR},\mathrm{BY},\mathrm{YB}\right\}\right) \end{array}$$

where ^*^ indicates convolution, $\theta \in \left\{0,\frac{\pi }{4},\frac{\pi }{2},\frac{3\pi }{4}\right\}$, and *g*_*e*, θ_(*x, y*) is the even-symmetric Gabor filter defined as:

(5) $$\begin{array}{l} g_{\;e,\theta }\left(x,y\right)=\exp\left(-\frac{{x^{\prime }}^{2}+\gamma^{2}{y^{\prime }}^{2}}{2\sigma^{2}}\right)\cos\left(\omega x^{\prime }\right) \end{array}$$

(6) $$\begin{array}{l} x^{\prime }=x\cos\theta +y\sin\theta ,\;y^{\prime }=-x\sin\theta +y\cos\theta \end{array}$$

where γ is the spatial aspect ratio, σ is the standard deviation, and ω is the spatial frequency. These definitions are the same as in Russell et al. (2014).

Then, the opponency signal ${F_{opp}}_{\theta }^{k}\left(x,y\right)$ is calculated by taking differences between the output of orthogonal Gabor filters of the same scale as:

(7) $$\begin{array}{l} {F_{opp}}_{\theta ,C}^{k}\left(x,\;y\right)=\left\lfloor F_{\theta ,C}^{k}\left(x,\;y\right)-F_{\theta +\frac{\pi }{2},C}^{k}\left(x,\;y\right)\right\rfloor \end{array}$$

This represents inhibition between simple cells which have antagonistic preferences of orientation.

Complex cells with larger RFs receive the simple cell outputs. We model this as a max-pooling operation. Determining the most appropriate size of the max-pooling filter is not trivial. We therefore let ourselves be guided by biological plausibility. According to Hubel (1988), receptive field size of complex cells in the fovea of macaque monkeys is about six times larger than that of simple cells, but the optimum stimulus width is about the same. In our model, the receptive field size of the Gabor filter (a model of simple cell) is set to about 13 pixels with about 3 pixels width of the center excitatory region as in Russell et al. (2014). We therefore set the diameter of the max pooling operation to 15 pixels.

We propose three types of texture channels: the spatial pooling channel, cross-scale channel, and cross-orientation channel. The first, spatial pooling channel $J_{1}$, is similar to one used in a previous model (Uejima et al., 2018), but max-pooling is used instead of the Gaussian filter. This can be written as:

(8) $$\begin{array}{l} J_{1,\theta ,C}^{k}\left(x,\;y\right)=MAX\left[{F_{opp}}_{\theta ,C}^{k}\left(x,\;y\right)\right] \end{array}$$

where MAX[·] is max-pooling operation within a circular area. Each pooling operation is applied adjacently (i.e., the stride is set to 1).

This process can occur within V1 cortex, but V2 may be involved as described in Biologically-Plausible Saliency Model. In the classical view, layer 4B of V1, which mainly includes magnocellular information, projects to the thick stripes of V2, and layer 2/3, which mainly originated from the parvo-/konio- pathway, projects to the pale stripes of V2. Recent studies have, however, suggested that the connections are more mixed (Nassi and Callaway, 2009). After V2, we assume our algorithm is processed in the ventral pathway which is responsible mainly for form/color perception and object recognition. The output of the texture detection stage is fed to the proto-object detection mechanism including edge detection. See Supplementary Materials for details.

Cross-scale channel $J_{2}$ and cross-orientation channel $J_{3}$ emulate the processes that are apparently done in V2 (Freeman et al., 2013). A cross-scale channel emphasizes where similar small elements [sometimes called “textons” (Julesz, 1981)] are repeated at different scales. This is calculated by:

(9) $$\begin{array}{l} J_{2,\theta ,C}^{k}\left(x,\;y\right)=MAX\left[{F_{opp}}_{\theta ,C}^{k}\left(x,\;y\right)\;\times \;{F_{opp}}_{\theta ,C}^{k+2}\left(x,\;y\right)\right]\\ \left(k\in \left\{1,2,3,4\right\}\;\right)\\ J_{2,\theta ,C}^{k}\left(x,\;y\right)=MAX\left[{F_{opp}}_{\theta ,C}^{k}\left(x,\;y\right)\;\times \;{F_{opp}}_{\theta ,C}^{k-4}\left(x,\;y\right)\right]\\ \left(k\in \left\{5,6,7\right\}\right) \end{array}$$

where × means pixel-wise product after resizing a map to the other map's size. The cross-orientation channel is

(10) $$\begin{array}{l} J_{3,\left(\theta ,\phi \right),C}^{k}\left(x,\;y\right)=MAX\left[{F_{opp}}_{\theta ,C}^{k}\left(x,\;y\right)\;\times \;{F_{opp}}_{\phi ,C}^{k}\left(x,\;y\right)\right]\\ \left(\theta =0,\frac{1}{2}\pi \;\right)\\ \left(\phi =\frac{1}{4}\pi ,\frac{3}{4}\pi \right) \end{array}$$

**Figure 2:**
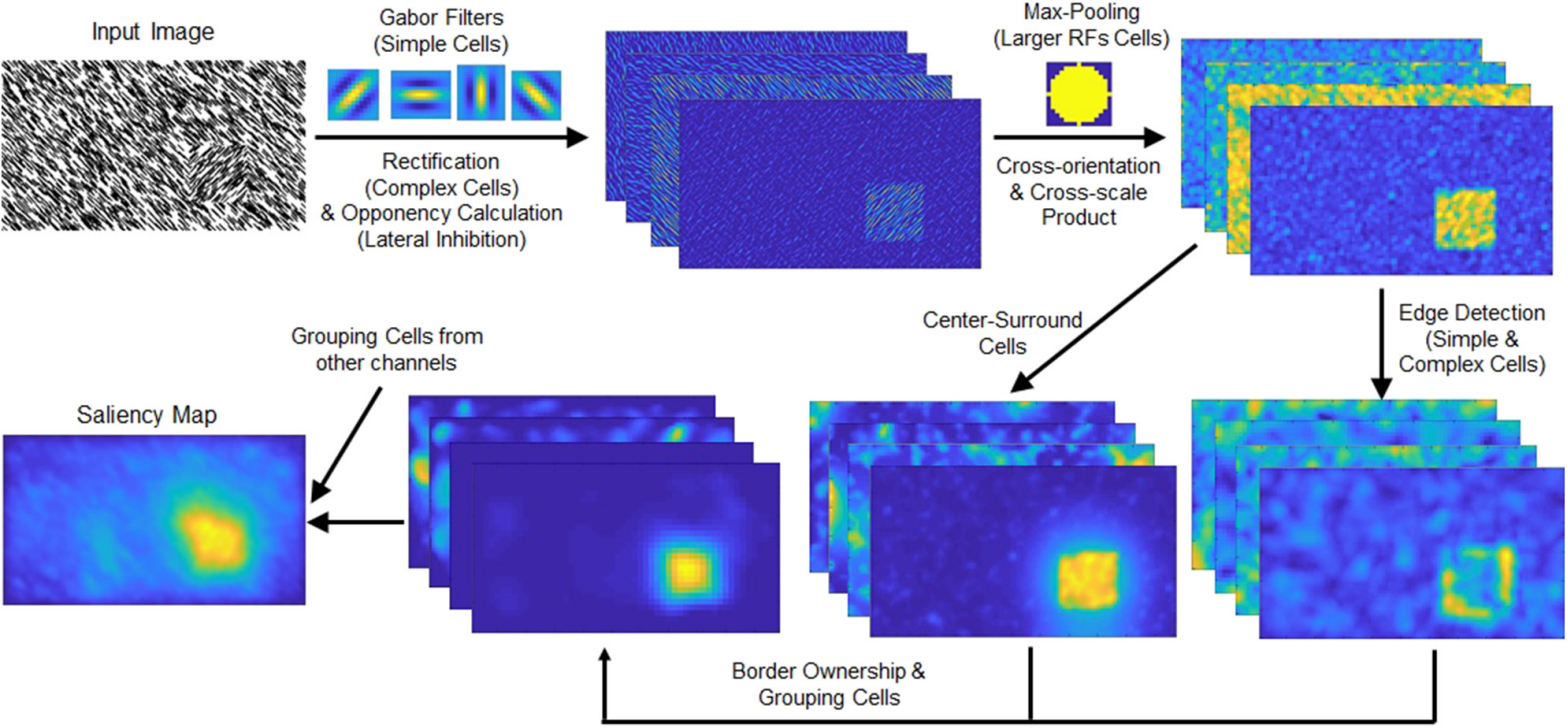
(Top row) Schematic of the spatial pooling texture channel. The intensity and color opponency maps (only intensity map shown) of the input image are convolved with Gabor filters of four orientations. The responses are rectified and fed to a max-pooling operation. This process combines the Gabor filter responses and highlights the texture-defined object (located in the lower right part of the input image). (Bottom row) Schematic chart of the grouping algorithm. The texture channel output is processed through the edge detection and center-surround cells. The grouping cells' output is summed with the other channels, intensity, color, and orientation.

An outline of the texture channel generation is drawn in the upper row of Figure 2 (only the spatial pooling channel is shown). As shown in the figure, a max pooling mechanism works to spatially combine the features extracted by Gabor filters and to form proto-objects. A similar approach using rectified Gabor filter outputs has been used in prior visual saliency research (Imamoglu and Lin, 2011). However, that approach did not consider the Gaussian filter as a method to combine texture elements, as we have done here, nor did it use max pooling.

The texture channels' outputs are shrunk after the max pooling process such that center-surround cells can cover various sizes of objects in the grouping algorithm. Because second-order texture mechanisms seem to operate at a spatial scale eight times or more coarse than first-order features (Zhou and Baker, 1993; Sutter et al., 1995), maps are resized to 1/7.5 of their original size. After resizing, the max-pooling operation with 15-pixel diameter becomes 2-pixel diameter, and center-surround cells which operate in up to five octaves to cover coarser second-order features.

Note that for computational efficiency, we implement the image pyramid for all features by keeping fixed sizes for all filters and max pooling operations, and scale the input image. This imparts scale invariance in all feature channels.

**Figure 3:**
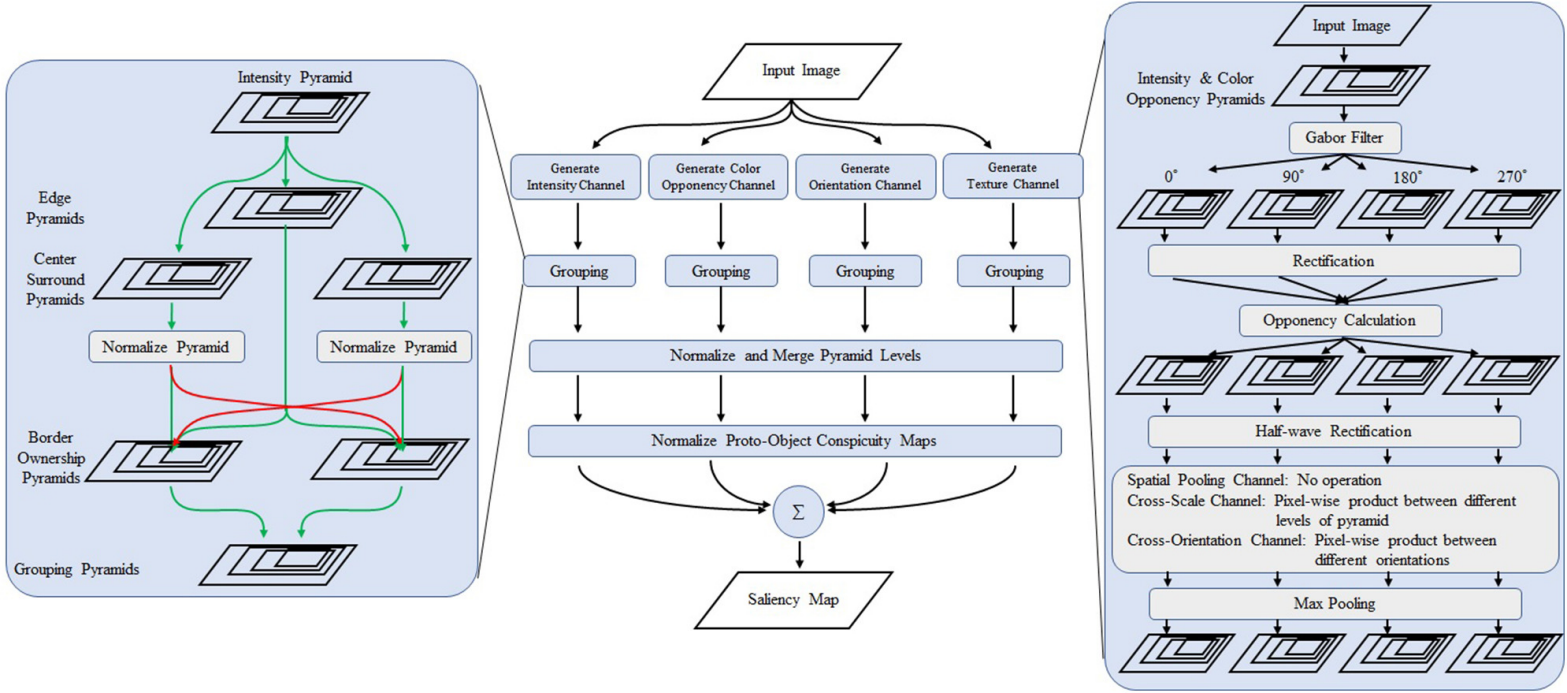
Overview of the proposed model. The center part shows the overall structure of the model. The left box is a detailed view of the grouping algorithm. The right box shows the schematics of the proposed texture channels. The first stage consists of Gabor filters of 0, 90, 180, and 270°. The opponency function is calculated from the output of the perpendicular filters. After rectification, cross-scale and cross-orientation products are obtained followed by max pooling operation whose receptive field is larger than the first stage Gabor filter is applied. Modified from Uejima et al. (2018) with permission.

The outputs of texture feature channels are fed to the proto-object mechanism described in Supplementary Material. A schematic view of the grouping process is shown in the bottom row of Figure 2, and an overview of the model is shown in Figure 3. As Figure 2 shows, the proposed texture channel enables the formation of a “boundary” at the interface between differently textured regions (the regions do not originally have borders), so that the model can detect these boundaries, assign their ownership to either figure or ground in the region, and ultimately group them into proto-objects before their saliency is determined.

We propose two type of saliency models. The proposed model 1 includes all channels of low-level features (intensity, color, and orientation) and three texture channels. The proposed model 2 incorporates only the spatial-pooling texture channel. See Supplementary Material for details.

### Experimental Setup

#### Saliency Dataset

To validate our algorithm, we determine its performance by its ability to predict human eye fixations on public datasets: TORONTO (Bruce and Tsotsos, 2005), CAT2000 training dataset (Borji and Itti, 2015; Bylinskii et al., 2019), MIT1003 (Judd et al., 2009), and FIGRIM (Bylinskii et al., 2015). These datasets are briefly described below.

##### TORONTO dataset

The TORONTO dataset includes 120 color images and fixation data from 20 subjects. Images were presented to the subjects for 4 s each. The resolution of the images is 681 pixels by 511 pixels. The images were shown on a 21-inch CRT display which was positioned 0.75 m from the subjects.

##### CAT2000 training dataset

The CAT2000 training dataset includes 2,000 images from 20 categories. All images are high definition resolution, 1,920 pixels by 1,080 pixels. We removed gray margins that were added to the images to preserve the aspect ratio before using them in the proto-object based models because they generate artifacts at the margins. 120 observers (24 observers per image) were placed 106 cm away from a 42-inch monitor such that scenes subtended ~45.5° × 31° of visual angle. Each image was shown for 5 s.

##### MIT1003 dataset

The MIT1003 dataset consists of 1,003 images with fixation data from 15 users. The longer dimension of each image is 1,024 pixels and the other dimension varies from 405 to 1,024 pixels. The images were presented for 3 s. with a 1 s. gray screen in between. The display showing the images was 19 inches and the distance between the display and users was about 2 feet.

##### FIGRIM dataset

The FIGRIM dataset is not collected from saliency experiments but from memory experiments. The dataset has two types of images, target images and filler images. A target image was shown first to subjects, followed by non-target (filler) images and the target image. The subjects had to answer whether each image was the target or not. Images remained on the screen for 2 s. Though this is a memory task and different from the free-looking tasks of typical saliency experiments, what the subjects had to do is similar: investigating the images for a few seconds.

Images in the dataset have 1,000 pixels by 1,000 pixels resolution and were presented on a 19-inch CRT monitor. The chinrest mount for the subject was set 22 inches away from the display. Forty subjects participated in the experiment. The dataset has 630 target images and 2,157 filler images.

##### Adjustment of stimulus resolution

Since we are comparing human fixation data with model output, it is important to match the resolution of the filters in the model with images observed by the subjects. If the model image resolution does not mimic that of the human visual system, the comparison between the model output and the human fixation data will not be meaningful. Hence, we determined the optimal image size for the model that mimics the human visual system for the datasets.

A reasonable way to adjust the input image resolution is to scale it so that the degree of visual angle (DVA) for the dataset equals the DVA of the algorithm. The DVA of the dataset is determined by the experimental setup: the actual screen size, distance between the screen and subject, and image resolution. The DVA of the model is determined by the size of filters. In our algorithm, the wavelength of the finest Gabor filter is set at 4 pixels. On the other hand, the finest primate simple cell's center excitation region width is a few minutes of arc (Hubel, 1988). Thus, the filter range in the algorithm can be thought to be from 30 pixels per degree or more.

For the CAT2000, MIT1003, and FIGRIM datasets, the DVAs are 30s pixels per degree, and they match our algorithm. On the other hand, the DVA of TORONTO dataset is about 20 pixels per degree, which means the dataset's resolution is too low for our algorithm. Thus, the images of TORONTO dataset are scaled to twice their original sizes before being fed to our algorithm.

#### Evaluation Score

Evaluating how well saliency models predict human fixations is not a simple problem. In the field of saliency prediction, several metrics have been proposed and used to evaluate the models, with different properties and characteristics (Wilming et al., 2011; Bylinskii et al., 2019). The published datasets provide two types of human fixation data: fixation locations and fixation maps. The fixation locations are binary value maps which indicate whether the observers fixated at each location or not. The fixation maps are scalar-value map obtained by blurring fixation locations by a 2D Gaussian kernel whose sigma is typically set to one degree of visual angle (see also Effect of Blurring the Saliency Map).

Recently, Kümmerer et al. proposed to redefine saliency maps and fixations in a probabilistic framework and simplify the evaluation problem (Kümmerer et al., 2015, 2018). One problem with this formulation is that it is necessary to convert existing models to probabilistic ones. In the current paper, we therefore use classical methods such as blurring saliency maps and add distance-to-center weight as written in Effect of Blurring the Saliency Map and Considering Center-Bias. Below we describe the metrics we use in this work.

Pearson's Correlation Coefficient (CC) is a statistical metric to measure how correlated two variables are. The CC between two variables, X and Y, is computed from their covariance σ(*X, Y*) and standard deviation σ(*X*) and σ(*Y*) as follows:

(11) $$\begin{array}{l} CC=\frac{\sigma \left(X,\;Y\right)}{\sigma \left(X\right)\;\times \;\sigma \left(Y\right)} \end{array}$$

The CC of two uncorrelated variables is 0, and negative CC means inverse correlation.

The similarity metric (SIM) is a metric for scalar-value maps. To compute it, first two maps X and Y (e.g., a human fixation map and a saliency map) are normalized so that $\underset{i}{\sum }X_{i}=\underset{i}{\sum }Y_{i}=1$ where *i* indicates pixel locations. Then, SIM is calculated as:

(12) $$\begin{array}{l} SIM=\underset{i}{\sum }\min\left(X_{i},\;Y_{i}\right) \end{array}$$

A SIM value of 0 means there is no overlap between two maps, and identical maps give 1.

The normalized scan-path saliency (NSS) is a location-based metric. To calculate the NSS, each saliency map is linearly normalized to have zero mean and unit standard deviation. The NSS is the mean value of the normalized saliency map at the fixation locations (Peters et al., 2005). An attractive property of the NSS is that it penalizes false positives and false negatives equally (Bylinskii et al., 2019). As a baseline, the chance value of NSS is 0, and the larger the NSS value the better.

The Kullback-Leibler divergence (KLD) is a scalar-value based metric from information theory. To compute the KLD, the human fixation map (called Y in the following equation) and the saliency map (called X) are treated as probability distributions. KLD indicates the loss of information when the saliency map is used to approximate the human fixation data. Smaller KLD signifies better performance. KLD can be computed as:

(13) $$\begin{array}{l} KLD=\underset{i}{\sum }Y_{i}\log\left(\epsilon +\frac{Y_{i}}{\epsilon +X_{i}}\right) \end{array}$$

where ϵ is a small regularization constant.

The shuffled AUC (sAUC) is a modified version of the area under the ROC curve (AUC). The receiver operating characteristic (ROC) measures the ratio of true positives and false positives at various thresholds. The saliency map is treated as a binary classifier to separate positive from negative samples. Thresholds are varied, and the ROC curve is determined by true positive rate and false positive rate at varying thresholds. The area under the curve is the result. The difference between sAUC and standard AUC is that sAUC samples negative points to calculate the false positive rate from fixation locations of other images instead of uniformly random locations. This compensates for the center-bias effect (and other systematic biases present in all images) where human fixation shows significantly higher density at the center of the display regardless of image content (Borji and Itti, 2012; Borji et al., 2013b; Zhang and Sclaroff, 2016). As a baseline, the sAUC indicates 0.5 by chance.

These metrics were calculated using the published code from Bylinskii et al. (2019).

#### Model Comparison

Numerous saliency models have been proposed by a number of researchers (Borji et al., 2013b). We have compared our proposed models with representative models; AIM (Bruce and Tsotsos, 2005), BMS2016 (Zhang and Sclaroff, 2016), GBVS (Harel et al., 2007), HouNIPS (Hou and Zhang, 2008), HouPAMI (Hou et al., 2012), Itti et al. originally from Itti et al. (1998) but we used the latest code from Harel et al. (2007), Judd et al. (Judd et al., 2009), SUN (Zhang et al., 2008), and DeepGaze2 (Kümmerer et al., 2016).

Unlike other saliency models, including our proposed models, the DeepGaze2 model is designed to calculate an output as a probability distribution which needs to be converted to saliency maps. Each map is optimized for each metric that is used for comparison to human subject fixation data (Kümmerer et al., 2015). We used the *pysaliency toolbox* (https://github.com/matthias-k/pysaliency) to compute the saliency maps for each metric, except the similarity measures that need nonlinear optimization to convert to maps. Instead, we used the saliency map optimized for CC to compute the similarity. Also, the DeepGaze2 model can incorporate center-bias effects where humans tend to fixate at the center of the display as a prior distribution. In this paper, we used a uniform distribution as a prior distribution for the DeepGaze2 and incorporated the center-bias in the same way as for the other models, as stated in section Considering Center-Bias.

We also evaluated human inter-observer performance, which shows inter-observer consistency and can be thought of as the upper-bound performance of each dataset. To compute inter-observer performance, we used three-fold evaluation: the subjects are divided into three groups in each dataset, and one group is treated as “ground truth” (test) and the other two groups are treated as a prediction model (training). This calculation is repeated three times with swapping groups, and then results are averaged. The center-bias compensation described in Considering Center-Bias is not applied to the inter-observer data since they already have the center-bias by their definition.

#### Effect of Blurring the Saliency Map

One of the difficulties of comparing saliency models is that blurring (smoothing) the saliency maps can largely affect the results (Borji and Itti, 2012; Hou et al., 2012). Typically, the blurring is applied to approximate the effect of the sampling error of the eye tracker used in the human study that generated the human fixation maps. Here, we applied a Gaussian kernel with various sizes to the saliency map of each model. The sigma of the Gaussian kernel was varied from sigma values from 0.01 to 0.20 of the image widths in steps of 0.01. For the sAUC metric, we used smaller sigma values up to 0.08 of the image widths because of sAUC's preference of smaller sigma. See the Supplementary Material for the effect of blurring. To compare the models, we chose the best result for each model and metric.

#### Considering Center-Bias

As mentioned in Evaluation Score, human fixations have significant center-bias, and all metrics, except the sAUC, are affected by it. This can be compensated to some extent by differentially weighting salience relative to the distance from the image center [e.g., by a Gaussian (Parkhurst et al., 2002; Parkhurst and Niebur, 2003)]. In this study, we have used distance-to-center (DTC) re-weighting to compare different models in the same way as the NSS calculation of Zhang and Sclaroff (2016). The DTC map is calculated as:

(14) $$\begin{array}{l} DTC\left(i,\;j\right)=1-\frac{\sqrt{{\left(i-\frac{H}{2}\right)}^{2}+{\left(j-\frac{W}{2}\right)}^{2}}}{\sqrt{{\left(\frac{H}{2}\right)}^{2}+{\left(\frac{W}{2}\right)}^{2}}} \end{array}$$

where *i* and *j* are the row and column index and *H* and *W* are the height and width of the stimulus image. The saliency map blurred by the best size Gaussian kernel is pixel-wise multiplied with the DTC map, and it is used to calculate the respective metric. The DTC re-weighting has not been applied to calculate the sAUC metric because sAUC itself compensates for the center-bias.

## Results

**Figure 4:**
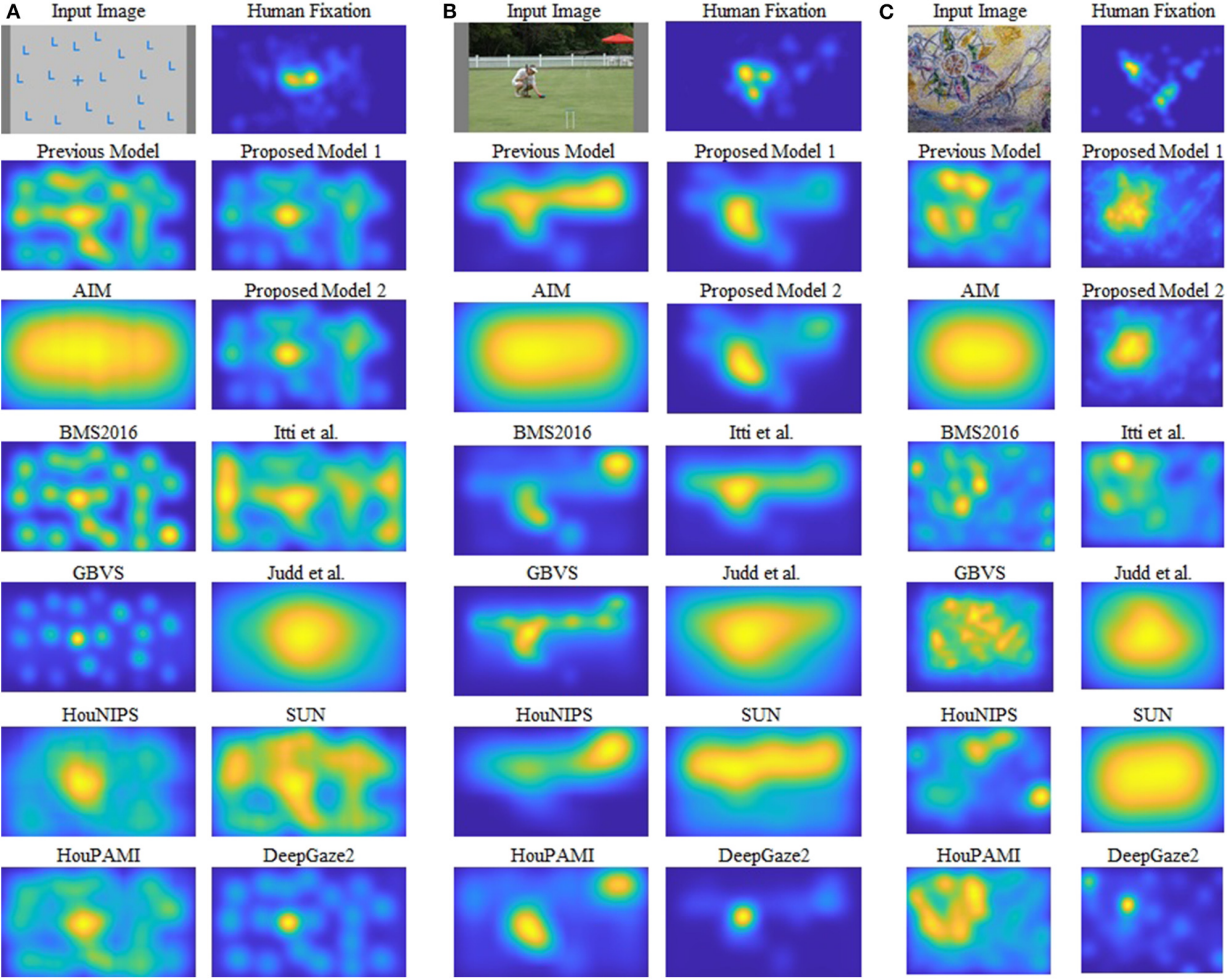
Examples of the results. Input images and human fixations are from the CAT2000 training dataset and the MIT1003. The saliency maps are blurred by a Gaussian kernel whose sigma is set to the best value for the similarity metric, and the DTC re-weighting is applied. **(A–C)** Each show one image and the corresponding human fixation map in the top row, and outputs of different models in other rows. See text for details.

As shown in Figure 1, our models can segregate a figure defined by a texture formed by oriented bars from a background, while all other models cannot clearly detect it. Figure 4 shows saliency maps of our models and other saliency models including the previous proto-object based model (without texture channels), AIM (Bruce and Tsotsos, 2005), BMS2016 (Zhang and Sclaroff, 2016), GBVS (Harel et al., 2007), HouNIPS (Hou and Zhang, 2008), HouPAMI (Hou et al., 2012), Itti et al. originally from Itti et al. (1998) but we used the latest code from Harel et al. (2007), Judd et al. (Judd et al., 2009), SUN (Zhang et al., 2008), and DeepGaze2 (Kümmerer et al., 2016). The saliency maps of each model are blurred by Gaussian filters whose standard deviations are set as resulting the best SIM (similarity) metrics for each model as described in Effect of Blurring the Saliency Map. They are also re-weighted for DTC as described in Considering Center-Bias. Images and human fixations in the figure are from the CAT2000 training dataset and the MIT1003 dataset. We include non-blurred saliency maps in Supplementary Materials since blurring sometimes makes it difficult to see where the models are activated.

Figure 4A shows a quite simple example: the image shows one “+” among many “L” s, and subjects tend to look at the unique “+”. This kind of stimulus can be discriminated by center-surround (or oriented) RFs with different sizes as pointed out by Bergen and Adelson (1988). The saliency of this stimulus, therefore, can be predicted by the previous proto-object based model (Russell et al., 2014), as well as most of the other models. On the other hand, an input such as Figure 4B is a rather complex scene and illuminates both advantages and limitations of our proposed model. There is a person in the foreground, with a fence and a colorful parasol in the background. Human observers tend to fixate preferentially the person's face and the left hand. There is likely a strong top-down component in this selection since the person is using the left hand to interact with two colored balls next to it (the balls are difficult to see in the small image shown in this figure, a larger version of the image that shows them more clearly is provided in Supplementary Material). The previous Russell et al. model showed high saliency at the fence and the person in the foreground, and possibly the parasol. Our new models capture objectness but they see a (proto-)object and not a person, and they fail to selectively attribute saliency to the face and the hands. This, of course, is expected since our models do not have semantic knowledge and, in particular, do not have any mechanism to detect faces or hands, features known to be of high behavioral relevance to humans. The same lack of specificity to these features is found in all except one model. This exception is DeepGaze2, a DNN-based model, which shows high saliency at the face location. It also fails to predict fixations to the hands, because, we speculate, it was not trained with image data that included hands as objects that are relevant in the training data set.

Figure 4C shows mosaic tiles of patterns and persons. The subjects mainly fixate at the center of a circular pattern in the upper-left and a face in the lower-right. Our proposed models successfully signal high saliency at the (center or boundary) of the pattern, but fail to predict fixations at the face, for the same reason as discussed above. The other models, except DeepGaze2, show weak or no saliency at the pattern center. The result of DeepGaze2 is similar to that of our proposed models on this image: high saliency at the center of the pattern, but very limited if any saliency is attributed to the face. Interestingly, DeepGaze2 seems to be exquisitely sensitive to faces: The DeepGaze2 map has a secondary maximum in the upper-left corner which seems to correspond to faces in the image. They were not fixated by human subjects, probably due to their high eccentricity.

Tables 1A–D shows the quantitative performance comparison among the models on each dataset. The MIT1003 dataset was used to train DeepGaze2, and this model's performance in Table 1C cannot be compared with the other models because the training data were also used for testing. Inter-observer performance (i.e., the theoretically best possibly performance) is also shown as a reference. As stated in Model Comparison, inter-observer metrics were calculated by three-fold validation. Therefore, the mean number of fixations in ground truth used for the inter-observer is only one-third of all fixations. Some metrics, especially KLD, are affected by the number of fixations and show worse results (Wilming et al., 2011). The reason is that, to calculate inter-observer performance, human fixation data (ground truth) needs to be separated into different groups which will in general have different numbers of fixations. The number of fixations affects the density of the maps, with small numbers of fixations resulting in sparse maps and large numbers of fixations in more distributed maps. KLD being a comparison of probability distributions, this may result in artifactual differences between model-generated maps and ground truth. On the other hand, NSS is, by definition, insensitive to the number of fixations and can be thought of as upper-bound predictability.

**Table 1:** Quantitative evaluation of saliency models on four datasets.

	**Proposed**		**Proposed**		**Previous**		**AIM**	**BMS2016**	**GBVS**	**HouNIPS**	**HouPAMI**	**Itti et al**.	**Judd et al**.	**SUN**	**Deep**	**Inter-**
	**Model 1**		**Model 2**		**Model**										**Gaze2**	**Observer**
**(A) EVALUATION SCORES ON TORONTO DATASET**																
NSS	1.739	5	1.753	4	1.517*^†^	8	1.190*^†^	1.925*^†^	1.644*^†^	1.455*^†^	1.776	1.551*^†^	1.442*^†^	1.213*^†^	*2*.199*^†^	2.438
CC	0.641	5	0.646	3	0.558*^†^	8	0.481*^†^	0.681*^†^	0.617*^†^	0.529*^†^	0.643	0.585*^†^	0.557*^†^	0.491*^†^	0.763*^†^	0.742
SIM	0.542*	3	0.548*^†^*	2	0.501*^†^	7	0.418*^†^	0.522*^†^	0.524*^†^	0.495*^†^	0.497*^†^	0.505*^†^	0.462*^†^	0.435*^†^	0.620*^†^	0.594
KLD	0.718	2	0.729	3	0.811*^†^	7	1.037*^†^	0.735	0.829*^†^	0.987*^†^	0.803*^†^	0.799*^†^	0.916*^†^	1.008*^†^	0.530*^†^	0.910
sAUC	0.691	5	0.686	4	0.670*^†^	8	0.692	0.724*^†^	0.638*^†^	0.673^†^	0.708*^†^	0.659*^†^	0.619*^†^	0.660*^†^	0.756*^†^	0.734
**(B) EVALUATION SCORES ON CAT2000 TRAINING DATASET**																
NSS	1.599*	3	1.623*^†^*	2	1.552*^†^	6	1.482*^†^	1.554*^†^	1.538*^†^	1.452*^†^	1.511*^†^	1.502*^†^	1.593*	1.496*^†^	1.717*^†^	2.447
CC	0.670*	3	0.677*^†^*	2	0.656*^†^	5	0.632*^†^	0.654*^†^	0.649*^†^	0.617*^†^	0.640*^†^	0.637*^†^	0.662*^†^	0.636*^†^	0.686*^†^	0.845
SIM	0.605*	3	0.613*^†^*	2	0.582*^†^	5	0.531*^†^	0.586*^†^	0.580*^†^	0.544*^†^	0.557*^†^	0.560*^†^	0.543*^†^	0.548*^†^	0.614*^†^*	0.704
KLD	0.593	3	0.585*^†^*	2	0.625*^†^	6	0.711*^†^	0.619*^†^	0.617*^†^	0.712*^†^	0.649*^†^	0.647*^†^	0.669*^†^	0.680*^†^	0.537*^†^	0.546
sAUC	0.596	7	0.595	8	0.599	5	0.611*^†^	0.622*^†^	0.582*^†^	0.591	0.609*^†^	0.591^†^	0.565*^†^	0.597	0.641*^†^	0.616
**(C) EVALUATION SCORES ON MIT1003 DATASET**																
NSS	1.426*	4	1.441*^†^*	2	1.258*^†^	7	1.167*^†^	1.606*^†^	1.439	1.186*^†^	1.235*^†^	1.332*^†^	1.345*^†^	1.179*^†^	2.565	2.806
CC	0.460*	4	0.469*^†^*	2	0.420*^†^	7	0.396*^†^	0.503*^†^	0.466	0.394*^†^	0.416*^†^	0.427*^†^	0.442*^†^	0.400*^†^	0.725	0.747
SIM	0.400*	2	0.408*^†^*	1	0.374*^†^	6	0.325*^†^	0.391*^†^	0.398*	0.374*^†^	0.362*^†^	0.376*^†^	0.349*^†^	0.333*^†^	0.529	0.591
KLD	1.198	3	1.198	2	1.274*^†^	6	1.451*^†^	1.175*^†^	1.203	1.398*^†^	1.293*^†^	1.256*^†^	1.343*^†^	1.393*^†^	0.738	0.988
sAUC	0.644*	6	0.633^†^	9	0.641*	7	0.675*^†^	0.702*^†^	0.631^†^	0.636	0.665*^†^	0.644*	0.599*^†^	0.649*	0.782	0.778
**(D) EVALUATION SCORES ON FIGRIM DATASET**																
NSS	1.616*	5	1.682*^†^*	3	1.587*^†^	8	1.507*^†^	1.588*^†^	1.633^†^	1.397*^†^	1.550*^†^	1.593*^†^	1.737*^†^	1.521*^†^	*2*.025*^†^	2.864
CC	0.530*	4	0.540*^†^*	2	0.521*^†^	7	0.497*^†^	0.522*^†^	0.536*^†^*	0.458*^†^	0.510*^†^	0.524*^†^	0.521*^†^	0.502*^†^	0.614*^†^	0.740
SIM	0.432*	3	0.446*^†^*	2	0.402*^†^	7	0.357*^†^	0.412*^†^	0.423*^†^	0.383*^†^	0.371*^†^	0.410*^†^	0.386*^†^	0.364*^†^	0.479*^†^	0.575
KLD	1.045*	3	1.012*^†^*	2	1.144*^†^	7	1.287*^†^	1.095*^†^	1.072*^†^	1.241*^†^	1.232*^†^	1.110*^†^	1.153*^†^	1.260*^†^	0.904*^†^	0.988
sAUC	0.630*	5	0.624^†^	7	0.621^†^	8	0.640*^†^	0.664*^†^	0.612*^†^	0.619	0.640*^†^	0.618^†^	0.582*^†^	0.629*	0.705*^†^	0.662
**(E) EVALUATION SCORES ON Black AND WHITE CATEGORY OF CAT2000 TRAINING DATASET**																
NSS	1.716*	3	1.799*^†^*	2	1.653*^†^	5	1.588*^†^	1.646*^†^	1.648*^†^	1.540*^†^	1.619*^†^	1.619*^†^	1.696*	1.581*^†^	2.140*^†^	
CC	0.644*	3	0.664*^†^*	2	0.627*^†^	5	0.605*^†^	0.623*^†^	0.623*^†^	0.585*^†^	0.614*^†^	0.613*^†^	0.629*^†^	0.601*^†^	0.705*^†^	
SIM	0.571*	3	0.593*^†^*	2	0.543*^†^	5	0.495*^†^	0.538*^†^	0.544*^†^	0.495*^†^	0.517*^†^	0.527*^†^	0.506*^†^	0.493*^†^	0.604*^†^*	
KLD	0.671*	3	0.638*^†^*	2	0.713*^†^	5	0.827*^†^	0.719*^†^	0.700*	0.803*^†^	0.756*^†^	0.737*^†^	0.775*^†^	0.820*^†^	0.567*^†^	
sAUC	0.624	3	0.620	6	0.620	7	0.622	0.627	0.602*^†^	0.589*^†^	0.623	0.604*^†^	0.585*^†^	0.588*^†^	0.669*^†^	

*The best three scores for each metric are underlined. The numbers written in the right column in the proto-object based models (three models form the left) are the orders among the models. Daggers and asterisks indicate statistical significance of the difference (paired t-test, p ≤ 0.05) from the proposed model 1 and 2, respectively. Inter-Observer scores are also shown as references. Some metrics of the inter-observer model show worse results because of fewer fixations. The results of the MIT1003 dataset of DeepGaze2 are gray-hatched because the dataset is used to train DeepGaze2 and cannot be compared with other models*.

**Figure 5:**
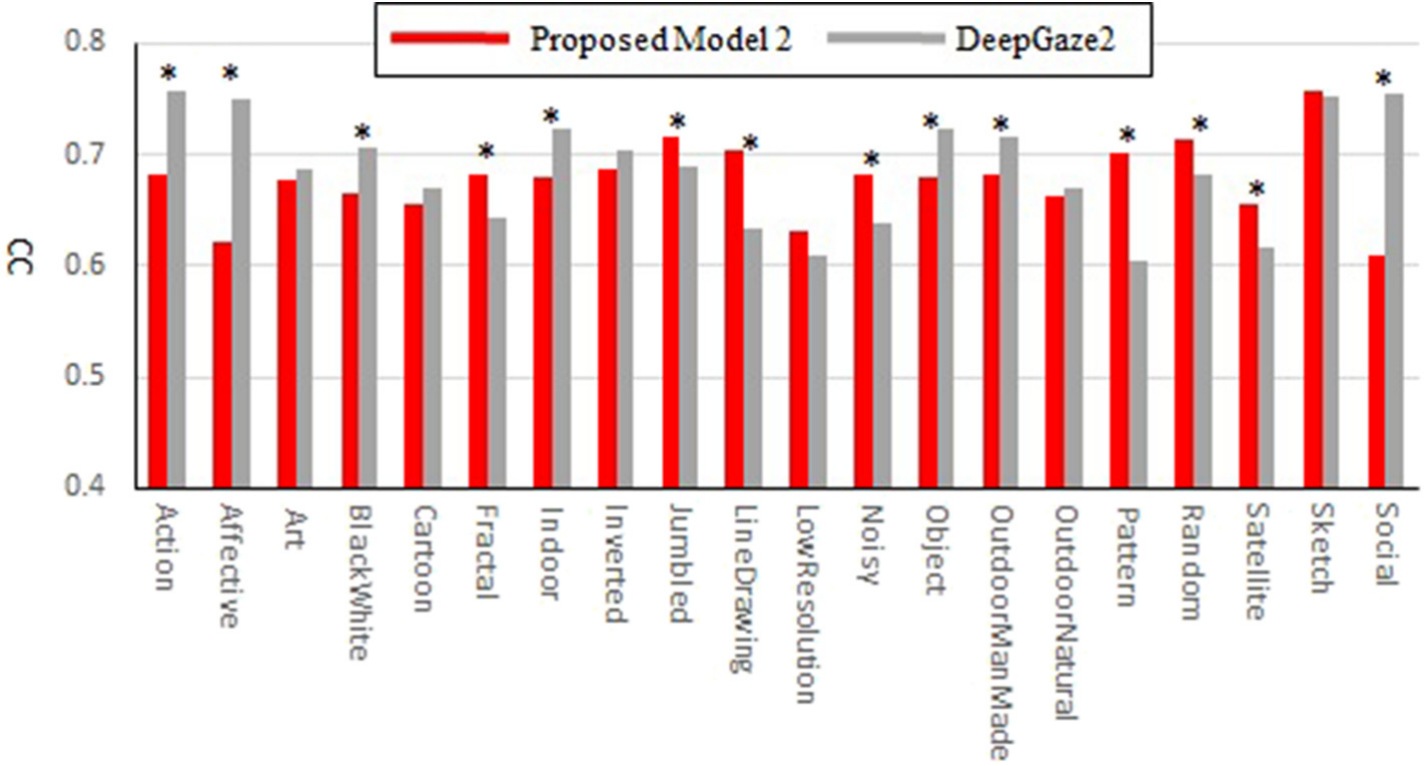
Correlation coefficients comparison between the proposed model 2 and DeepGaze2 by categories of the CAT2000 training dataset. Asterisks indicate statistical significance of the difference in corresponding categories (paired *t*-test, *p* ≤ 0.05).

**Figure 6:**
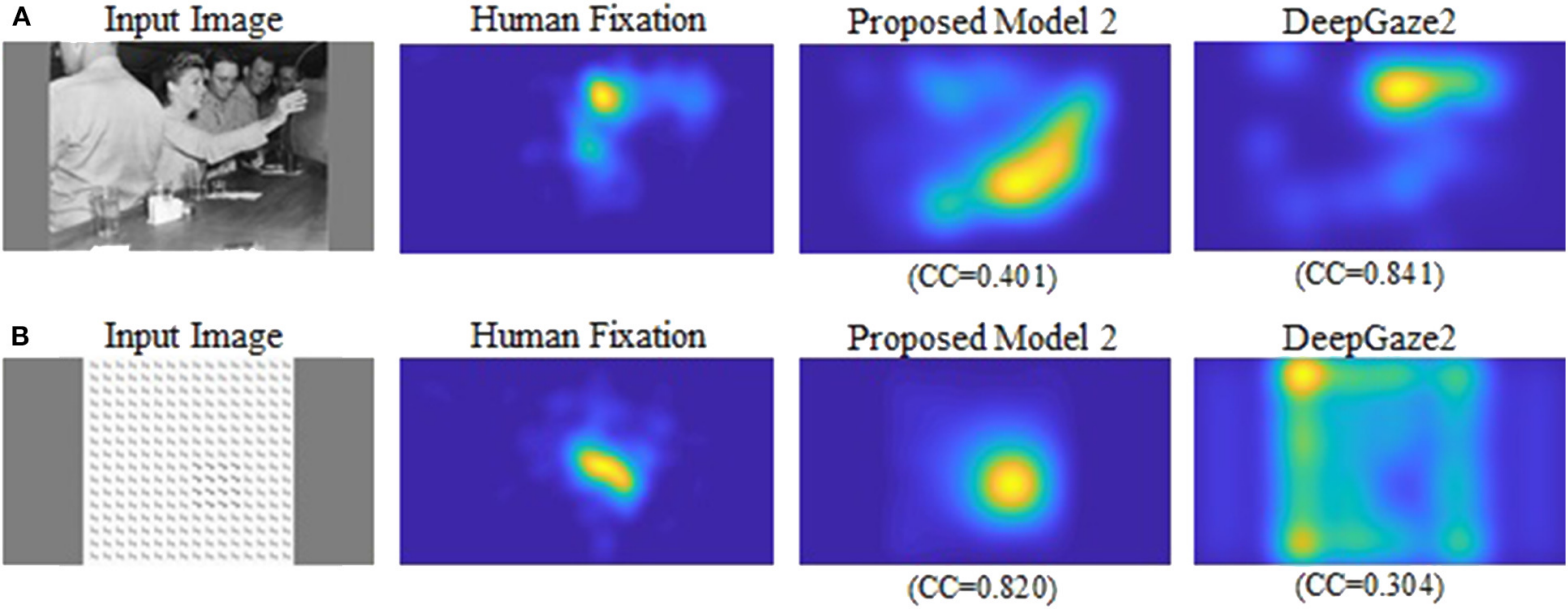
**(A)** Image for which DeepGaze2 showed the best CC relative to the proposed model 2 in the Social category. **(B)** Image for which the proposed model 2 showed the best CC relative to DeepGaze2 in the Pattern category.

Our new two models outperformed the previous model for all datasets and metrics. Comparison between the two new models shows that model 2 has better performance for most of the metrics. Also, in most comparisons it is ranked in the top three among all models. Of particular interest is DeepGaze2, the only DNN network in our comparison set. It shows significantly better results than all other models including both of ours, as expected from the MIT saliency benchmark (Bylinskii et al., 2009). One likely reason is that, by design, DeepGaze2 is trained on image features that are known to have behavioral relevance to humans (e.g., faces) while our models use only hard-coded constructs based on Gestalt principles. However, DeepGaze2 sometimes misses salient simple and low-level features like the balls in Figure 4B. To better understand the relative merits of our models and DeepGaze2, we compared the models separately on different categories of the CAT2000 training dataset in Figure 5. Out of 14 categories with significant differences between our model 2 and DeepGaze2, the latter works better on seven. These include the Social, Affective, and Action images which typically include many images with persons and faces. Figure 6A shows the image in the Social category for which DeepGaze2 produced the best CC relative to our model 2. In agreement with human fixations, DeepGaze2 placed high saliency on the faces and less on other objects, such as the glasses on the counter. In contrast, our model 2 showed high values on the counter, which were of relatively little apparent interest to humans, and only very limited weight at the face locations. On the other hand, our model 2 worked significantly better than DeepGaze2 on the other image classes, including the Pattern, LineDrawing, and Random categories which mainly consist of synthesized or artificial images. Figure 6B shows the image where our model 2 showed the best performance relative to DeepGaze2 in the Pattern category. DeepGaze2 failed to predict fixations on the group of slightly modified patterns, while our method predicted these fixations. It were, however, not only over-simplified or highly abstract images where our model outperformed DeepGaze2. It also had significantly better performance on satellite images. We believe that the reason is that these images can be described by image statistics inherent in natural scenes (including Gestalt laws) which are implemented in our models. We surmise that satellite images are absent or strongly underrepresented in the dataset used to train VGG-19, and thus DeepGaze2, leading to a lower performance on these images. Our model also seems more robust to noise, as shown by its comparative advantage in the Noisy class.

Most types of texture are discriminable even in grayscale images. Texture perception may be of ecological relevance particularly in low-light conditions since the only light receptors usable in such conditions (rods) do not produce a color perception. To investigate the effect of the lack of color, we have summarized the result for the Black and White category of the CAT2000 dataset which contains 100 gray-scale (mostly outdoor) images in Table 1E. For these images, our two models are in 2nd and 3rd place except for the sAUC metrics (overall best performance is shown by DeepGaze2).

**Figure 7:**
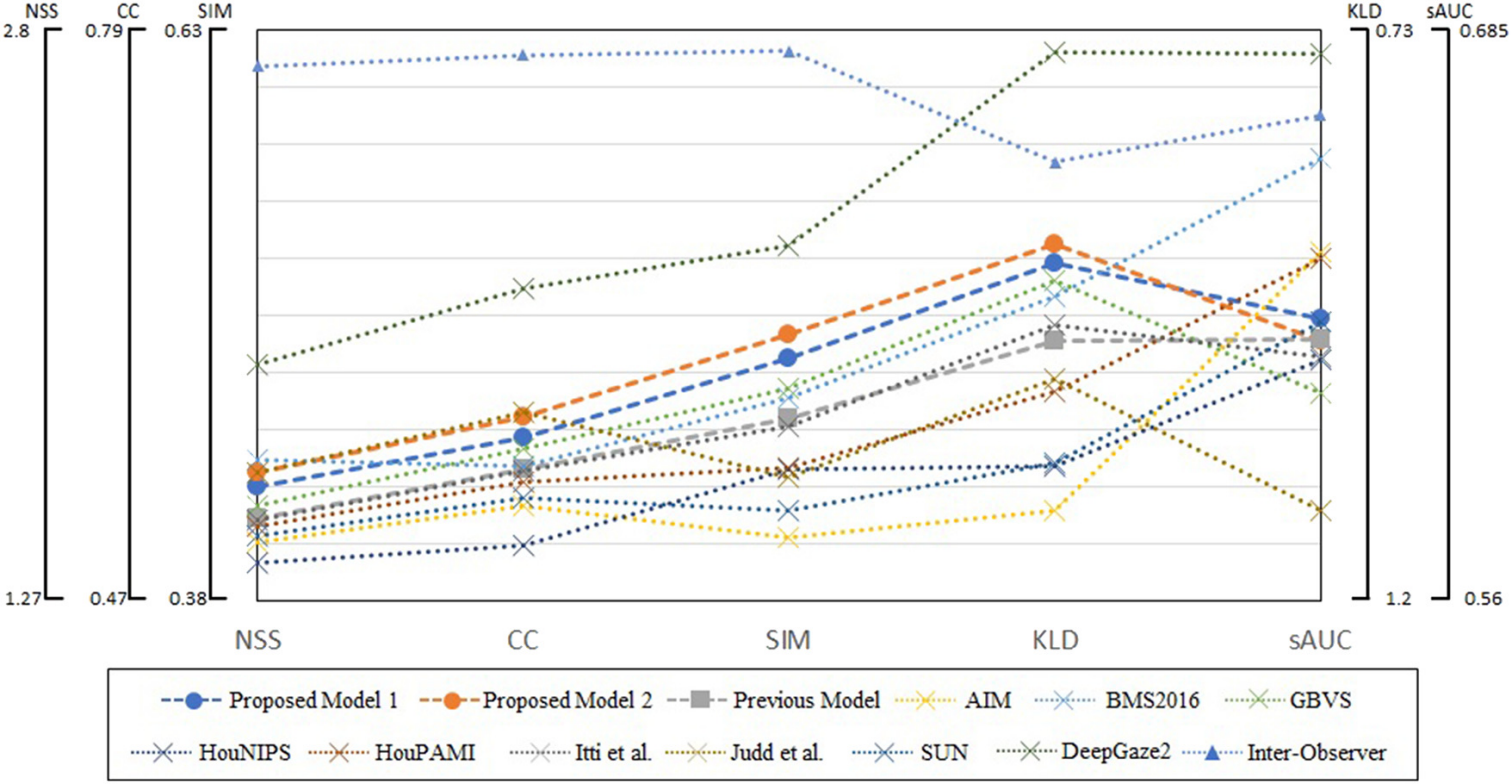
Summary of evaluation scores on four datasets. Circle markers indicate the proposed models, the square markers the previous proto-object based model, and the cross markers show other saliency models. The inter-observer data are shown by the triangle markers. The metrics of DeepGaze2 was computed without the MIT1003 dataset since it was a part of its training data.

We summarized evaluation scores on all four datasets in Figure 7. The metrics are averages on 5,910 images, and parameters of blurring Gaussian kernel were chosen as those showed the best performances for each model and metric. The scores of DeepGaze2 were calculated by excluding MIT1003 since it was used to train the model. The presented models constantly show significant improvement from the conventional proto-object based model and in most cases better performance than other methods on most stimuli, with one exception. This exception is DeepGaze2 which clearly outperforms all other models, by all measures.

The proposed algorithms are implemented using MATLAB (The Mathworks, Natick, MA). Execution time is about 13 min for the proposed model 1 and 3.5 min for the proposed model 2 on a PC with a Ryzen 7 1700X 3.4 GHz processor and 32 GB memory for a 1,920 × 1,080-pixel image.

## Discussion

Our models show improvement over the original proto-object based saliency model in predicting human fixations which already performed very well. Through four datasets, our model 2 shows the second performance for two metrics (SIM and KLD) and third best for CC as shown in Figure 7. This means our model is close to the best at predicting human fixations, with the exception of the DNN-based model, DeepGaze2 which outclasses all models by a wide margin.

But what is the core mechanism of our two models, and why do they work so well? To simplify the problem, let us focus on the simpler (and better performing) one, model 2. The model extracts features by multi-scale Gabor filters and then applies a proto-object detection algorithm. This means that the model assumes that objects have localized specific oriented spectral components (as features), and a foreground object can be detected if its components are sufficiently different from other objects and a background. In other words, each oriented spectral component works as a carrier, and an object is an envelope defined by an ensemble of carriers. The previous proto-object based model used simple intensity (or color) as a feature instead of oriented spectral components. Thus, it does not work well when a foreground object has shadows or is not discriminable by mean intensity/color contrast. In that case, the previous model tends to focus on a part of the object or other objects that are lighter or darker than the whole of the foreground as shown in Figure 4B.

The premise of our proposed models is similar to HouPAMI (Hou et al., 2012), which supposes that the foreground is spatially sparse and the background is spectrally sparse, even though the implementations of the two models are completely different. In our model, the proto-object detection algorithm works to find blobs that are spatially sparse, and it prefers single or a few blobs, while omitting features with numerous peaks (i.e., dense features). In the spectral domain, we assume that the foreground has different components (i.e., different frequency or orientation) from the background, and an object that is dense in the frequency domain has more chance of surviving the normalization process. As a result, our models have a tendency to put saliency on an object that is spatially sparse and spectrally dense. It seems that our implementation of Gestalt principles by biologically quite realistic mechanisms is functionally similar to the explicit implementation of the principles formulated by Hou et al. (2012).

It is somewhat difficult to understand why our proposed model 2 shows better performance than our model 1 which includes low-feature channels, cross-scale texture and cross-orientation texture channels. The absence of intensity and color channels in model 2 means it does not have non-orientation selective feature detectors. However, isotropic stimuli, such as a circle, can be detected by the Gabor filter. For instance, large and small circles can be discriminated by large and small Gabor filters. On the other hand, the reason why the cross-scale and cross-orientation features does not improve performance very much is not easy to understand. One possibility is that such mid-level features are necessary to discriminate complex textures, but do not attract people's gaze in free-viewing experiments.

Table 1E shows that the texture channel works particularly well on images without color information. We believe that this indicates that when texture is the predominant structure in the image, our biologically plausible model with the texture channel performs as well or better than the computational state-of-the-art models, except for DeepGaze2. This also means our methods may be applicable to other intensity-based images such as infrared videos, synthetic-aperture radar pictures etc.

Recent studies show that DNNs achieve high performance in object classification but they heavily depend on the texture of objects rather than their shape, while humans put importance on the object shape (Baker et al., 2018; Geirhos et al., 2018). This is reasonable because in static scenes, texture cues are more robust than the shape which changes and is modified by changes of the direction from which pictures were taken from, especially when the training data include various views for each object. However, texture cues are subject to their own distortions (e.g., the influence of speckled illumination under a tree canopy). Humans rely much more on shape than DNNs (Geirhos et al., 2018) which may also have to do with the fact that their visual system is “trained” in a dynamic world in which shape changes smoothly and typically predictably, rather than in world in which unrelated static scenes are presented in random order, which would correspond to typical DNN training paradigms. We surmise that one of the results of shape dominance is that humans recognize texture-defined objects such as the input image of Figure 1 even without obvious borders. Therefore, it is natural to think humans treat texture features as a cue not only to recognize an object, but also to determine its shape. For these reasons, our shape based (proto-object based) model can capture the shape of texture-defined object thanks to the combination of Gabor filters, max-pooling, and grouping algorithm as shown in Figure 2.

The only DNN included among the models tested, DeepGaze2, showed significantly better results than any other. As described in Biologically-Plausible Saliency Model, we believe the successful results of DeepGaze2 are due to the fact that it is built on the VGG-19 network, which was originally trained for object recognition. DeepGaze2 worked especially well when input images included people and faces, but not as well on synthetic or artificial images. This is not surprising because the datasets used to train and tune DeepGaze2 (ImageNet, SALICON, and MIT1003) include few synthetic images, and many faces and people. On the other hand, we can say it generalizes surprisingly well given that almost no synthetic images were used in its training. A concern about DNN-based models is that the mechanisms underlying model performance are notoriously difficult to understand. Related is their enormous number of free parameters, more than 143 million for VGG-19 alone. While, in the right context, such over-parametrization can be beneficial (Soltanolkotabi et al., 2019), it carries substantial computational cost. In a recent study, Thompson et al. (2020) argue that the truly impressive progress of DNNs over the last 8 years will be severely curtailed by the prohibitive cost required, in monetary, environmental and other terms. They estimate that, for instance in the field of image classification, lowering the classification error on ImageNet rate from 11.5% (current state of the art) to 5% (close to human performance) will increase the training cost by a factor of 10^13^, to a staggering 10^19^ US Dollars, on the order of hundred-thousand times the estimate of the 2020 world GDP, with a concomitant environmental impact. Clearly, a different approach is needed. Given that biology has solved the problem, at the cost of a power consumption of about 20 W, identifying mechanisms used by biological grains seems highly promising. Therefore, the main purpose of our research is to understand and model cortical mechanisms for attention and scene understanding. For this reason, we take a first principles, constructive approach, which may lead to better understating of biological solutions of these problems.

The current study focuses on bottom-up attention only. Our proposed models include only early stages of visual processing that are thought as pre-attentive. This means that fixation prediction of our models is valid only for relatively short periods of observation (typically a few seconds) under free-viewing or non-specified tasks. Also, the models are mostly constructed to be compatible with primate neurophysiology. Visual systems of humans and macaques have similar anatomical organization (Sereno et al., 1995), and the early visual processing is functionally homologous (De Valois et al., 1974). In addition, the V4 region, which is essential for form and color perception and attention, is similar in both species (Gallant et al., 2000). While they have many similarities, there are also differences. For instance, Einhäuser et al. (2006) claims that humans are attracted more by higher-level features than monkeys. In the current study, the models have been extended by incorporating mid-level feature, i.e., texture, but still lack higher-order level features such as human bodies, animals, faces, and written text, which generally attract human fixations [an example where a simple face detector is included in saliency computations is Cerf et al. (2008)]. Another factor which can affect human attention is familiarity. Studies have shown that unfamiliar target patterns are easier to find than familiar patterns during visual search (Wang et al., 1994; Greene and Rayner, 2001). Unfortunately, such effects are difficult to incorporate in our models, among other reasons because they lack a memory component.

One physiologically controversial point of our proposed models may be that their fundamental elements are neurons tuned to both color and orientation. This differs from the feature integration theory of Treisman and Gelade (1980) in which these combinations are not used as basic elements of pre-attentive processes (Treisman and Gelade, 1980). Neurophysiological studies, however, found that a sizable fraction of V1 neurons code color and orientation jointly (Hanazawa et al., 2000; Friedman et al., 2003; Johnson et al., 2008; Garg et al., 2019). The functional reason for this coding scheme may be that it reflects the statistics of natural scenes. Indeed, independent component analysis shows that the majority of independent components are oriented red-green and blue-yellow filters (most of the other components are oriented intensity filters) (Tailor et al., 2000). We also note that, although we formulate our models in terms of receptive fields of “neurons,” all our conclusions remain valid when the models are, instead, defined in terms of functional spatio-chromal units with the receptive field structures that we use. Another limitation is that our models lose high-resolution information due to a max-pooling operation. When we look at the input image of Figure 1, we can effortlessly tell the boundary of the pattern-defined object in close-to pixel-wise manner although the edge cannot be defined by each pixel (the oriented lines need several pixels to be defined). The max-pooling operation dilates the figure information and its precise shape is lost. Pixel-wise accuracy is not important for saliency prediction because human fixation measurements are not precise either. It is, however, important for other applications such as detection of salient objects. How the brain solves this problem is not clear, but recurrent processing between V1 and V2 and possibly higher areas may contribute to it as proposed in high-resolution buffer theory (Lee and Mumford, 2003).

Dealing with center-bias is one of the difficulties when evaluating visual saliency research. Whether the well-known center preference of human observers should be included in an algorithm or not depends on the purpose of a model. For instance, to predict how website viewers fixate contents, a model with center preference is suitable because the website is shown in a framed display. On the other hand, in truly “free-viewing” (frame less) environments, such as looking out over Times Square, it is less meaningful to consider center-bias. In this paper, we have used the DTC re-weighting process for the model comparison in an attempt to neutralize this bias.

The proposed model has the advantage of being a relatively simple extension of the proto-object saliency model which is based on biologically-plausible mechanisms, and supported by neurophysiological measurements (Zhou et al., 2000). In addition, the model does not need training data, although it needs parameter adjustment. Because of its feed-forward nature, it can also be readily implemented in hardware (Molin and Etienne-Cummings, 2015; Thakur et al., 2017; Narayanan et al., 2019). Although a strictly feed-forward architecture lacks biological plausibility, it should be noted that the model does mimic feedback pathways by opening feedback loops and reproducing them in a feedforward manner for computational efficiency.

## Conclusion

How texture is represented and processed in visual cortex is not well understood. Here, we have implemented a simple nonlinear texture feature detection mechanism inspired by Sutter et al. (1995), Mareschal and Baker (1998), Freeman et al. (2013) and incorporated it into a proto-object based saliency model. The performance of the model has been validated against human fixation data, and it shows substantial improvement over a previously published proto-object based saliency model without the texture feature channel, as well as over most state of the art models with other architectures against that we compared it.

Texture is a complicated feature, and the model implemented in this paper loses high-resolution information. Further ideas gleaned from biological systems will need to be added to the model to improve its performance in this respect. Predicting where humans fixate can enhance effectiveness of advertisements, traffic signs, virtual reality displays, and other applications. It may also be used to identify atypical texture patterns in nature, such as camouflage, because these models emulate how humans see and humans are very good at performing such texture segmentation tasks.

## Data Availability Statement

The original contributions generated for this study are included in the article/Supplementary Material, further inquiries can be directed to the corresponding author/s.

## Author Contributions

TU, EN, and RE-C: design, methodology, and analysis. TU: software and writing—original draft. RE-C: project administration and funding acquisition. EN and RE-C: supervision, writing—review, and editing. All authors contributed to the article and approved the submitted version.

## Conflict of Interest

The authors declare that the research was conducted in the absence of any commercial or financial relationships that could be construed as a potential conflict of interest.
