# Supplementary material for: Proto-Object Based Saliency Model With Texture Detection Channel
Source: Front Comput Neurosci. 2020 Sep 24;14:541581. doi: 10.3389/fncom.2020.541581 (PMC7541834; doi:10.3389/fncom.2020.541581)
Supplement: Supplementary file 1 [file Data_Sheet_1.docx]

Supplementary Material

# Proto-object based Model

## Low-level features

Low-level features are computed in the same way as in the Russell et al. model (Russell et al., 2014).

Intensity and color features are calculated as written in eqs. (1) – (3) in the main article but span a 10-level pyramid over five octaves instead of three octaves. Orientation features use the same map as intensity, but center-surround cells defined by Supplementary Material eq. (10) is replaced by even-symmetric Gabor filters.

## Feedforward Grouping Algorithm

The 10-level pyramid images of intensity, color, orientation, and the 7-level pyramid images of the texture channels are fed to the feedforward grouping process introduced by (Russell et al., 2014). We use the symbol $\beta^{k}(x, y)$ to represent the input pyramid image.

Edge detection and center-surround responses are the first operations of the grouping algorithm. Edge detection is implemented in terms of simple cells and complex cells (Adelson & Bergen, 1985; Morrone & Burr, 1988). For low-level features, these operations may correspond to computations in in layers 4C, 4B, and 2/3 of V1. For the texture channels, the edge detection of second-order feature may be done in V2. The simple cell responses are computed according to:

|  | $\mathcal{S}_{\boldsymbol{e,\theta}}^{\boldsymbol{k}}\left( \boldsymbol{x,y} \right)\boldsymbol{=}\boldsymbol{\beta}^{\boldsymbol{k}}\left( \boldsymbol{x,y} \right)\boldsymbol{*}\boldsymbol{g}_{\boldsymbol{e,\theta}}\boldsymbol{(x,y)}$  $\mathcal{S}_{\boldsymbol{o,\theta}}^{\boldsymbol{k}}\left( \boldsymbol{x,y} \right)\boldsymbol{=}\boldsymbol{\beta}^{\boldsymbol{k}}\left( \boldsymbol{x,y} \right)\boldsymbol{*}\boldsymbol{g}_{\boldsymbol{o,\theta}}\left( \boldsymbol{x,y} \right)$ |  |
| --- | --- | --- |

where $\mathcal{S}_{e,\theta}^{k}$ and $\mathcal{S}_{o,\theta}^{k}$ are the even and odd edge pyramids, and $g_{e,\theta}$ and $g_{o,\theta}$ are the even and odd Gabor filter kernels. The odd Gabor filter is defined as:

|  | $\boldsymbol{g}_{\boldsymbol{o,\theta}}\left( \boldsymbol{x,y} \right)\boldsymbol{=}\exp\left( \boldsymbol{-}\frac{{\boldsymbol{x}^{\boldsymbol{'}}}^{\boldsymbol{2}}\boldsymbol{+}\boldsymbol{\gamma}^{\boldsymbol{2}}{\boldsymbol{y}^{\boldsymbol{'}}}^{\boldsymbol{2}}}{\boldsymbol{2}\boldsymbol{\sigma}^{\boldsymbol{2}}} \right)\sin\left( \boldsymbol{\omega}\boldsymbol{x}^{\boldsymbol{'}} \right)$  $\boldsymbol{x}^{\boldsymbol{'}}\boldsymbol{=x}\cos\boldsymbol{\theta}\boldsymbol{+y}\mathbf{sin}\boldsymbol{\theta,}\boldsymbol{y}^{\boldsymbol{'}}\boldsymbol{=-x}\mathbf{sin}\boldsymbol{\theta+y}\cos\boldsymbol{\theta}$ |  |
| --- | --- | --- |

(see eqs. (5) and (6) for the definition of the even Gabor filters). The responses of the odd and even filters are combined using the energy representation as the complex cell at each angle $\theta$,

|  | $\mathcal{C}_{\boldsymbol{\theta}}^{\boldsymbol{k}}\left( \boldsymbol{x,y} \right)\boldsymbol{=}\sqrt{{\mathcal{S}_{\boldsymbol{e,\theta}}^{\boldsymbol{k}}\left( \boldsymbol{x,y} \right)}^{\boldsymbol{2}}\boldsymbol{+}{\mathcal{S}_{\boldsymbol{o,\theta}}^{\boldsymbol{k}}\left( \boldsymbol{x,y} \right)}^{\boldsymbol{2}}}$ |  |
| --- | --- | --- |

To infer whether the edges in $\mathcal{C}_{\theta}^{k}$ belong to figure or ground, a center-surround mechanism is used to retrieve the contextual information. The present model uses center-surround mechanisms of both polarities, with ON-center receptive fields identifying light objects on dark backgrounds and OFF-center ones detecting dark objects on light backgrounds. The center-surround response is calculated as:

|  | for the texture channels:  $\mathcal{CS}_{\boldsymbol{D}}^{\boldsymbol{k}}\left( \boldsymbol{x,y} \right)\boldsymbol{=}\sum_{\boldsymbol{j\geq k}} \mathcal{N}_{\mathbf{1}}\left( \left\lfloor\boldsymbol{\beta}^{\boldsymbol{j}}\left( \boldsymbol{x,y} \right)\boldsymbol{*}\boldsymbol{cs}_{\boldsymbol{off}}\left( \boldsymbol{x,y} \right) \right\rfloor\right)$ $\mathcal{CS}_{\boldsymbol{L}}^{\boldsymbol{k}}\left( \boldsymbol{x,y} \right)\boldsymbol{=}\sum_{\boldsymbol{j\geq k}} \mathcal{N}_{\mathbf{1}}\left( \left\lfloor\boldsymbol{\beta}^{\boldsymbol{j}}\left( \boldsymbol{x,y} \right)\boldsymbol{*}\boldsymbol{cs}_{\boldsymbol{on}}\left( \boldsymbol{x,y} \right) \right\rfloor\right)$  and for the other channels:  $\mathcal{CS}_{\boldsymbol{D}}^{\boldsymbol{k}}\left( \boldsymbol{x,y} \right)\boldsymbol{=}\mathcal{N}_{\mathbf{1}}\left( \left\lfloor\boldsymbol{\beta}^{\boldsymbol{k}}\left( \boldsymbol{x,y} \right)\boldsymbol{*}\boldsymbol{cs}_{\boldsymbol{off}}\left( \boldsymbol{x,y} \right) \right\rfloor\right)$ $\mathcal{CS}_{\boldsymbol{L}}^{\boldsymbol{k}}\left( \boldsymbol{x,y} \right)\boldsymbol{=}\mathcal{N}_{\mathbf{1}}\left( \left\lfloor\boldsymbol{\beta}^{\boldsymbol{k}}\left( \boldsymbol{x,y} \right)\boldsymbol{*}\boldsymbol{cs}_{\boldsymbol{on}}\left( \boldsymbol{x,y} \right) \right\rfloor\right)$ |  |
| --- | --- | --- |

where $\mathcal{CS}_{D}$ and $\mathcal{CS}_{L}$ form the dark and light object pyramids. Here, we note why multiscale center-surround cells are suitable for the texture channel. The size of the figure defined by texture does not have any relation with the size of texture elements (texton). The model, therefore, needs two types of scale pyramid: one for textons and one for the figures defined by textons.

${cs}_{off}$ and ${cs}_{on}$ are the OFF-center and ON-center center-surround filter kernel, respectively, generated by a difference of Gaussians as follows:

|  | $\boldsymbol{cs}_{\boldsymbol{on}}\left( \boldsymbol{x,y} \right)\boldsymbol{=}\frac{\boldsymbol{1}}{\boldsymbol{2}\boldsymbol{\pi}\boldsymbol{\sigma}_{\boldsymbol{i}}^{\boldsymbol{2}}}\boldsymbol{e}^{\boldsymbol{-}\frac{\boldsymbol{x}^{\boldsymbol{2}}\boldsymbol{+}\boldsymbol{y}^{\boldsymbol{2}}}{\boldsymbol{2}\boldsymbol{\sigma}_{\boldsymbol{i}}^{\boldsymbol{2}}}}\boldsymbol{-}\frac{\boldsymbol{1}}{\boldsymbol{2}\boldsymbol{\pi}\boldsymbol{\sigma}_{\boldsymbol{o}}^{\boldsymbol{2}}}\boldsymbol{e}^{\boldsymbol{-}\frac{\boldsymbol{x}^{\boldsymbol{2}}\boldsymbol{+}\boldsymbol{y}^{\boldsymbol{2}}}{\boldsymbol{2}\boldsymbol{\sigma}_{\boldsymbol{o}}^{\boldsymbol{2}}}}$ $\boldsymbol{cs}_{\boldsymbol{off}}\left( \boldsymbol{x,y} \right)\boldsymbol{=-}\frac{\boldsymbol{1}}{\boldsymbol{2}\boldsymbol{\pi}\boldsymbol{\sigma}_{\boldsymbol{i}}^{\boldsymbol{2}}}\boldsymbol{e}^{\boldsymbol{-}\frac{\boldsymbol{x}^{\boldsymbol{2}}\boldsymbol{+}\boldsymbol{y}^{\boldsymbol{2}}}{\boldsymbol{2}\boldsymbol{\sigma}_{\boldsymbol{i}}^{\boldsymbol{2}}}}\boldsymbol{+}\frac{\boldsymbol{1}}{\boldsymbol{2}\boldsymbol{\pi}\boldsymbol{\sigma}_{\boldsymbol{o}}^{\boldsymbol{2}}}\boldsymbol{e}^{\boldsymbol{-}\frac{\boldsymbol{x}^{\boldsymbol{2}}\boldsymbol{+}\boldsymbol{y}^{\boldsymbol{2}}}{\boldsymbol{2}\boldsymbol{\sigma}_{\boldsymbol{o}}^{\boldsymbol{2}}}}$ |  |
| --- | --- | --- |

where $\sigma_{i}$ is the standard deviation of the center (inner) Gaussian and $\sigma_{o}$ is the standard deviation of the surround (outer) Gaussian. These kernels are replaced by even Gabor filters to calculate the orientation channel.

$\mathcal{N}_{1}\left( \cdot\right)$ is the normalization operator used in the same way as by (Russell et al., 2014) which is similar to that from (Itti et al., 1998): The two members of the pair $\mathcal{CS}_{D}$ and $\mathcal{CS}_{L}$ are simultaneously normalized to the range of 0 to 10. Then the average of all local maxima, $\bar{m}$, is computed across both maps, and each map is multiplied by $\left( 10-\bar{m} \right)^{2}$. This normalization emphasizes the global maximum center-surround response and suppresses local maxima responses. Thus, it promotes grouping activity of maps with few proto-objects and lowers the grouping activity of maps with manyproto-objects.

Border ownership cells encode where the edges of an object belongs (rather than just oriented edges). These cells have been observed in monkey visual cortex (mainly V2) (Zhou et al., 2000). The firing activity of some of these cells is independent of contrast polarity. To simulate this, $\mathcal{B}_{\theta,L}^{k}$, the border ownership activity for a light object on a dark background and $\mathcal{B}_{\theta,D}^{k}$, one for a dark object on a light background are computed by:

| $\mathcal{B}_{\boldsymbol{\theta,L}}^{\boldsymbol{k}}\left( \boldsymbol{x,y} \right)\boldsymbol{=}\left\lfloor\mathcal{C}_{\boldsymbol{\theta}}^{\boldsymbol{k}}\left( \boldsymbol{x,y} \right)\boldsymbol{\times}\left( \boldsymbol{1+}\sum_{\boldsymbol{j\geq k}} \frac{\boldsymbol{1}}{\boldsymbol{2}^{\boldsymbol{j}}}\boldsymbol{v}_{\boldsymbol{\theta+\pi}}\left( \boldsymbol{x,y} \right)\mathcal{*C}\mathcal{S}_{\boldsymbol{L}}^{\boldsymbol{j}}\left( \boldsymbol{x,y} \right)\boldsymbol{-}\boldsymbol{w}_{\boldsymbol{opp}}\sum_{\boldsymbol{j\geq k}} \frac{\boldsymbol{1}}{\boldsymbol{2}^{\boldsymbol{j}}}\boldsymbol{v}_{\boldsymbol{\theta}}\left( \boldsymbol{x,y} \right)\mathcal{*C}\mathcal{S}_{\boldsymbol{D}}^{\boldsymbol{j}}\left( \boldsymbol{x,y} \right) \right) \right\rfloor$ | | |
| --- | --- | --- |
|  |  |  |
| $\mathcal{B}_{\boldsymbol{\theta,D}}^{\boldsymbol{k}}\left( \boldsymbol{x,y} \right)\boldsymbol{=}\left\lfloor\mathcal{C}_{\boldsymbol{\theta}}^{\boldsymbol{k}}\left( \boldsymbol{x,y} \right)\left( \boldsymbol{1+}\sum_{\boldsymbol{j\geq k}} \frac{\boldsymbol{1}}{\boldsymbol{2}^{\boldsymbol{j}}}\boldsymbol{v}_{\boldsymbol{\theta+\pi}}\left( \boldsymbol{x,y} \right)\mathcal{*C}\mathcal{S}_{\boldsymbol{D}}^{\boldsymbol{j}}\left( \boldsymbol{x,y} \right)\boldsymbol{-}\boldsymbol{w}_{\boldsymbol{opp}}\sum_{\boldsymbol{j\geq k}} \frac{\boldsymbol{1}}{\boldsymbol{2}^{\boldsymbol{j}}}\boldsymbol{v}_{\boldsymbol{\theta}}\left( \boldsymbol{x,y} \right)\mathcal{*C}\mathcal{S}_{\boldsymbol{L}}^{\boldsymbol{j}}\left( \boldsymbol{x,y} \right) \right) \right\rfloor$ | | |
|  |  |  |

where $w_{opp}$ is the weight of the inhibitory signal from the opposite polarity and $v$ is the von Mises distribution (Russell et al., 2014). Because the edge may be a part of the larger object, the $\mathcal{CS}$ activities of lower spatial scales are summed.

The activity of border ownership selective cells indicates which side of an edge belongs to a figure rather than the background. A technical difficulty is how boundaries of the finite-sized images are treated, because the approach taken may affect the saliency result substantially (Borji et al., 2013; Zhang et al., 2008). It is problematic to consider something near the boundary as a figure because there is no data outside the image. In (Russell et al., 2014), boundaries were zero-padded, i.e. the values of $\mathcal{C}\mathcal{S}_{L}^{k}$ and $\mathcal{C}\mathcal{S}_{D}^{k}$ are assumed to be zero outside of the input image in the border ownership activity calculation. We found that in our algorithm, zero-padding can show unexpected results because the inhibitory term (with $w_{opp}$) is set to zero during the border ownership calculation near the boundary. In the proposed model, $\mathcal{C}\mathcal{S}_{L}^{k}$ and $\mathcal{C}\mathcal{S}_{D}^{k}$ outside the image in the inhibitory term are therefore set to the maximum value of the input image instead of zero, which mitigates the contribution of the borders in the saliency computation.

As mentioned, in a sizable sub-population of cortical border ownership selective cells, border ownership responses are invariant to the contrast polarity of the figure and background. Thus, the responses of light and dark objects are combined to make them independent of figure-ground contrast polarity.

|  | $\mathcal{B}_{\boldsymbol{\theta}}^{\boldsymbol{k}}\left( \boldsymbol{x,y} \right)\boldsymbol{=}\mathcal{B}_{\boldsymbol{\theta,L}}^{\boldsymbol{k}}\left( \boldsymbol{x,y} \right)\boldsymbol{+}\mathcal{B}_{\boldsymbol{\theta,D}}^{\boldsymbol{k}}\boldsymbol{(x,y)}$ |  |
| --- | --- | --- |

At each pixel, multiple border ownership cells exist for each direction of border ownership. To determine which border a pixel belongs to, we use a winner-take-all algorithm between the response of a border ownership selective neuron $\mathcal{B}_{\theta}^{k}$ and its antagonist $\mathcal{B}_{\theta+\pi}^{k}$,

|  | ${\hat{\mathcal{B}}}^{\boldsymbol{k}}\boldsymbol{(x,y)=}\mathcal{B}_{\hat{\boldsymbol{\theta}}}^{\boldsymbol{k}}\boldsymbol{(x,y)}$ |  |
| --- | --- | --- |

where

|  | $\hat{\boldsymbol{\theta}}\boldsymbol{=}\mathbf{arg}\max_{\boldsymbol{\theta}} \left( \mathcal{B}_{\boldsymbol{\theta}}^{\boldsymbol{k}}\left( \boldsymbol{x,y} \right)\mathbf{-}\mathcal{B}_{\boldsymbol{\theta+\pi}}^{\boldsymbol{k}}\left( \boldsymbol{x,y} \right) \right)$ |  |
| --- | --- | --- |

As the final stage of the grouping algorithm, the “grouping” cell responses are calculated by summing the winning border ownership activity in an annular fashion (Russell et al., 2014). This operation may be implemented in V4, which is known to be important for form perception and visual attention but our results do not depend on which cortical area contains grouping cells. Their activity is computed by:

|  | $\mathcal{G}^{\boldsymbol{k}}\left( \boldsymbol{x,y} \right)\boldsymbol{=}\left\lfloor\left( \mathcal{B}_{\hat{\boldsymbol{\theta}}}^{\boldsymbol{k}}\left( \mathbf{x,y} \right)\boldsymbol{-}\boldsymbol{w}_{\boldsymbol{b}}\boldsymbol{\times}\mathcal{B}_{\hat{\boldsymbol{\theta}}\boldsymbol{+\pi}}^{\boldsymbol{k}}\left( \mathbf{x,y} \right) \right)\boldsymbol{*}\boldsymbol{v}_{\hat{\boldsymbol{\theta}}}\left( \boldsymbol{x,y} \right) \right\rfloor$ |  |
| --- | --- | --- |

where $w_{b}$ is the synaptic weight of the inhibitory signal from the $\mathcal{B}$ cell coding for the opposite direction of ownership.

## Normalization and Combining Channels

To compute the saliency map, each grouping cell response is normalized and combined in the same way as in (Russell et al., 2014). The normalizing operation, $\mathcal{N}_{2}\left( \cdot\right)$, is the same as for the center-surround responses: The input grouping cell response is normalized to the range 0 to 10, and divided by the average of the local maxima. This normalization process is designed to suppress maps that contain many local maxima of similar values, and to promote maps with few maxima (or even a global maximum) which are substantially higher than other maxima. The biological motivation behind this process is that it approximates cortical lateral inhibition mechanisms, in which neighboring similar features inhibit each other via global divisive inhibition mechanisms (Heeger, 1992, 1993).

To combine the data from multiple pyramids, a cross scale addition operation symbolized by $\bigoplus$ is used. This operation consists of scaling each map to spatial scale $k=8$ and then performing a pixel-wise addition.

The combined intensity channel, $\bar{\mathcal{I}}$, is calculated by:

|  | $\bar{\mathcal{I}}\mathbf{=}\boldsymbol{\bigoplus}_{\boldsymbol{k=1}}^{\boldsymbol{k=10}}\mathcal{N}_{\boldsymbol{2}}\left( \mathcal{G}_{\boldsymbol{I}}^{\boldsymbol{k}} \right)$ |  |
| --- | --- | --- |

For the color channel,

| $\bar{\mathcal{C}}\mathbf{=}\boldsymbol{\bigoplus}_{\boldsymbol{k=1}}^{\boldsymbol{k=10}}\left( \mathcal{N}_{\boldsymbol{2}}\left( \mathcal{G}_{\boldsymbol{RG}}^{\boldsymbol{k}} \right)\boldsymbol{+}\mathcal{N}_{\boldsymbol{2}}\left( \mathcal{G}_{\boldsymbol{GR}}^{\boldsymbol{k}} \right)\boldsymbol{+}\mathcal{N}_{\boldsymbol{2}}\left( \mathcal{G}_{\boldsymbol{BY}}^{\boldsymbol{k}} \right)\boldsymbol{+}\mathcal{N}_{\boldsymbol{2}}\left( \mathcal{G}_{\boldsymbol{YB}}^{\boldsymbol{k}} \right) \right)$ |  |
| --- | --- |

For the orientation channel,

|  | $\bar{\mathcal{O}}\boldsymbol{=}\sum_{\boldsymbol{\alpha\in}\left\{ \mathbf{0,}\boldsymbol{\pi}/\mathbf{4}\mathbf{,}\boldsymbol{\pi}/\mathbf{2}\mathbf{,}\boldsymbol{3\pi}/\mathbf{4} \right\}} \left( \boldsymbol{\bigoplus}_{\boldsymbol{k=1}}^{\boldsymbol{k=10}}\mathcal{N}_{\boldsymbol{2}}\left( \mathcal{G}_{\boldsymbol{O\alpha}}^{\boldsymbol{k}} \right) \right)$ |  |
| --- | --- | --- |

The texture channels for intensity map are computed by:

|  | ${\bar{\boldsymbol{T}}}_{\boldsymbol{I,1}}\boldsymbol{=}\sum_{\boldsymbol{\alpha\in}\left\{ \mathbf{0,}\boldsymbol{\pi}/\mathbf{4}\mathbf{,}\boldsymbol{\pi}/\mathbf{2}\mathbf{,}\boldsymbol{3\pi}/\mathbf{4} \right\}} \left( \boldsymbol{\bigoplus}_{\boldsymbol{k=1}}^{\boldsymbol{k=10}}\mathcal{N}_{\boldsymbol{2}}\left( \mathcal{G}_{\boldsymbol{T}_{\boldsymbol{1}}\boldsymbol{\alpha,I}}^{\boldsymbol{k}} \right) \right)$ |  |
| --- | --- | --- |
|  | ${\bar{\boldsymbol{T}}}_{\boldsymbol{I,2}}\boldsymbol{=}\sum_{\boldsymbol{\alpha\in}\left\{ \mathbf{0,}\boldsymbol{\pi}/\mathbf{4}\mathbf{,}\boldsymbol{\pi}/\mathbf{2}\mathbf{,}\boldsymbol{3\pi}/\mathbf{4} \right\}} \left( \boldsymbol{\bigoplus}_{\boldsymbol{k=1}}^{\boldsymbol{k=10}}\mathcal{N}_{\boldsymbol{2}}\left( \mathcal{G}_{\boldsymbol{T}_{\boldsymbol{2}}\boldsymbol{\alpha,I}}^{\boldsymbol{k}} \right) \right)$ |  |
|  | ${\bar{\boldsymbol{T}}}_{\boldsymbol{I,3}}\boldsymbol{=}\sum_{\boldsymbol{\alpha\in}\left\{ \mathbf{0,}\boldsymbol{\pi}/{\boldsymbol{2\},}}\boldsymbol{\beta=\{}\boldsymbol{\pi}/\boldsymbol{4}\mathbf{,}\boldsymbol{3\pi}/\mathbf{4} \right\}} \left( \boldsymbol{\bigoplus}_{\boldsymbol{k=1}}^{\boldsymbol{k=10}}\mathcal{N}_{\boldsymbol{2}}\left( \mathcal{G}_{\boldsymbol{T}_{\boldsymbol{3}}\left( \boldsymbol{\alpha,\beta} \right)\boldsymbol{,I}}^{\boldsymbol{k}} \right) \right)$ |  |

The texture channels for color opponency map are computed by:

|  | ${\bar{\boldsymbol{T}}}_{\boldsymbol{C,1}}\boldsymbol{=}\sum_{\boldsymbol{\alpha\in}\left\{ \mathbf{0,}\boldsymbol{\pi}/\mathbf{4}\mathbf{,}\boldsymbol{\pi}/\mathbf{2}\mathbf{,}\boldsymbol{3\pi}/\mathbf{4} \right\}} \sum_{\boldsymbol{\gamma\in}\left\{ \mathcal{RG,GR,BY,YB} \right\}} \left( \boldsymbol{\bigoplus}_{\boldsymbol{k=1}}^{\boldsymbol{k=10}}\mathcal{N}_{\boldsymbol{2}}\left( \mathcal{G}_{\boldsymbol{T}_{\boldsymbol{1}}\boldsymbol{\alpha,\gamma}}^{\boldsymbol{k}} \right) \right)$ |  |
| --- | --- | --- |
|  | ${\bar{\boldsymbol{T}}}_{\boldsymbol{C,2}}\boldsymbol{=}\sum_{\boldsymbol{\alpha\in}\left\{ \mathbf{0,}\boldsymbol{\pi}/\mathbf{4}\mathbf{,}\boldsymbol{\pi}/\mathbf{2}\mathbf{,}\boldsymbol{3\pi}/\mathbf{4} \right\}} \sum_{\boldsymbol{\gamma\in}\left\{ \mathcal{RG,GR,BY,YB} \right\}} \left( \boldsymbol{\bigoplus}_{\boldsymbol{k=1}}^{\boldsymbol{k=10}}\mathcal{N}_{\boldsymbol{2}}\left( \mathcal{G}_{\boldsymbol{T}_{\boldsymbol{2}}\boldsymbol{\alpha,\gamma}}^{\boldsymbol{k}} \right) \right)$ |  |
|  | ${\bar{\boldsymbol{T}}}_{\boldsymbol{C,3}}\boldsymbol{=}\sum_{\boldsymbol{\alpha\in}\left\{ \mathbf{0,}\boldsymbol{\pi}/{\boldsymbol{2\},}}\boldsymbol{\beta=\{}\boldsymbol{\pi}/\boldsymbol{4}\mathbf{,}\boldsymbol{3\pi}/\mathbf{4} \right\}} \sum_{\boldsymbol{\gamma\in}\left\{ \mathcal{RG,GR,BY,YB} \right\}} \left( \boldsymbol{\bigoplus}_{\boldsymbol{k=1}}^{\boldsymbol{k=10}}\mathcal{N}_{\boldsymbol{2}}\left( \mathcal{G}_{\boldsymbol{T}_{\boldsymbol{3}}\left( \boldsymbol{\alpha,\beta} \right)\boldsymbol{,I,\gamma}}^{\boldsymbol{k}} \right) \right)$ |  |

Finally, to generate the proto-object saliency map, these maps are normalized and combined:

|  | $\mathcal{S=}\boldsymbol{a}_{\boldsymbol{I}}\mathcal{N}\left( \bar{\mathcal{I}} \right)\boldsymbol{+}\boldsymbol{a}_{\boldsymbol{c}}\mathcal{N}\left( \bar{\mathcal{C}} \right)\boldsymbol{+}\boldsymbol{a}_{\boldsymbol{o}}\mathcal{N}\left( \bar{\mathcal{O}} \right)\boldsymbol{+}\sum_{\boldsymbol{n}} \boldsymbol{a}_{\boldsymbol{T}_{\boldsymbol{I}}\boldsymbol{,n}}\mathcal{N}\left( {\bar{\boldsymbol{T}}}_{\boldsymbol{I,n}} \right)\boldsymbol{+}\sum_{\boldsymbol{n}} \boldsymbol{a}_{\boldsymbol{T}_{\boldsymbol{C}}\boldsymbol{,n}}\mathcal{N}\left( {\bar{\boldsymbol{T}}}_{\boldsymbol{C,n}} \right)$ |  |
| --- | --- | --- |

The parameters $a_{x}$ determine the importance of each feature. Though they can be used to tune the model, we have used only the binary values 0 and 1 to select or deselect channels in the computation of the saliency maps. Here, we use three sets of parameters which are described in Supplementary Table 1. The previous model has only low-level features: intensity, color, and orientation. This is very similar to the model of (Russell et al., 2014) but includes an improvement in the border ownership calculation which is described in 3.3. The proposed model 1 has all features described above, including texture features. The proposed model 2 has only the spatial pooling texture channel.

Supplementary Table 1 Parameters for the two proposed models and the previous model (Russel et al 2014)


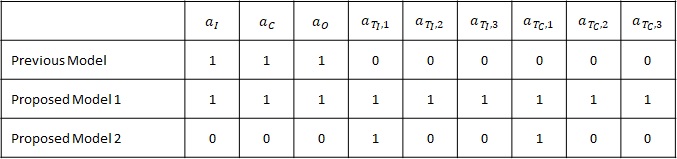


# Effect of Blurring

As describe in the main article, we applied a2D Gaussian kernel to the saliency maps of the models since some models generate relatively sparse maps and others denser ones. The best performance after blurring with the best standard deviations for each model and metric is shown in the main article. Here, we show how blurring affects metrics for each model in Supplementary Figure 1. The shown metrics are averages on four datasets, except for DeepGaze2 which uses only three datasets because the MIT1003 dataset was used for training. The data do not include distance-to-center re-weighting described in the article.


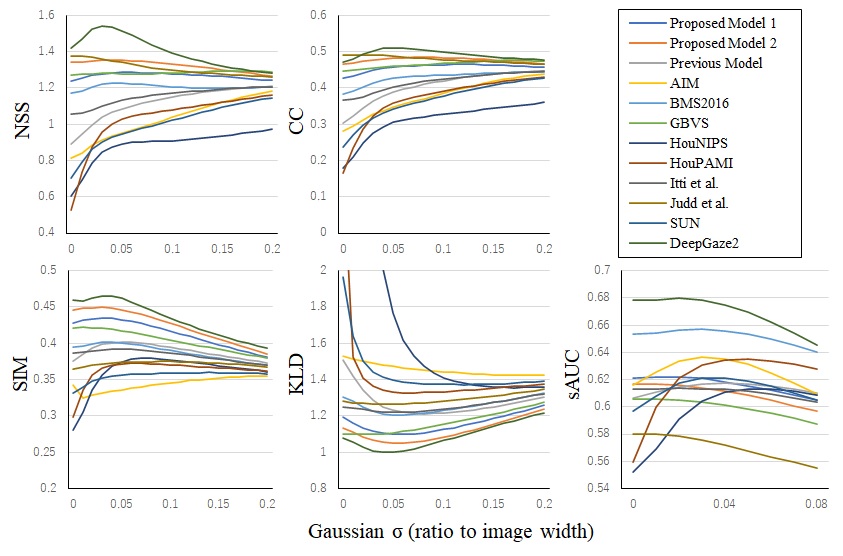


Supplementary Figure 1. Effect of blurring saliency maps on metrics.

# Non-blurred saliency maps

Fig. 4 of the main article shows some examples of saliency maps. But blurring sometimes make it difficult to see where models put saliency on. Supplementary Figure 2 shows non-blurred saliency maps overlapped on input images. The center-bias of human fixations is removed by subtracting a center-bias model which approximates the average of all human fixation maps with two 2D Gaussian distributions.


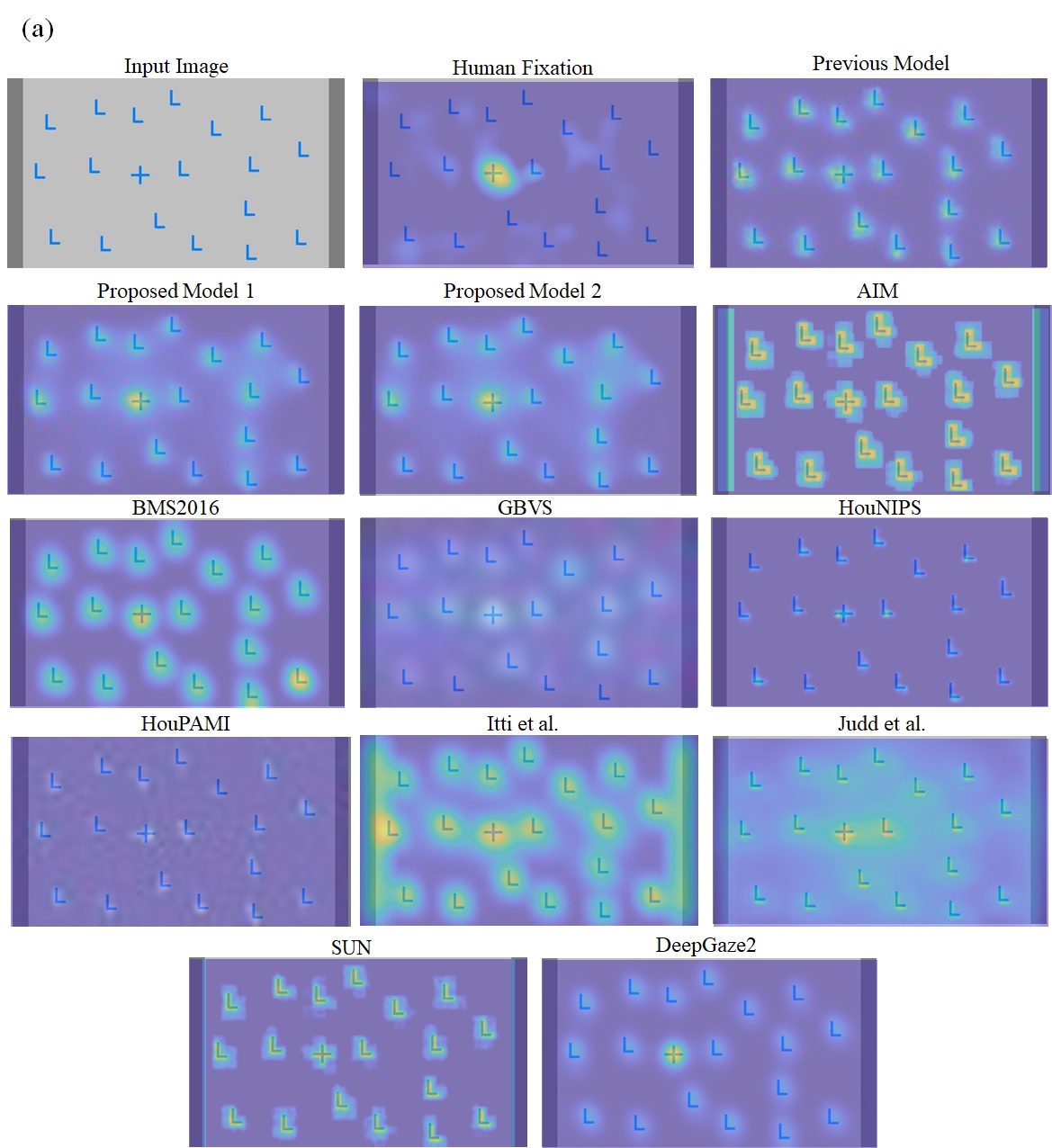


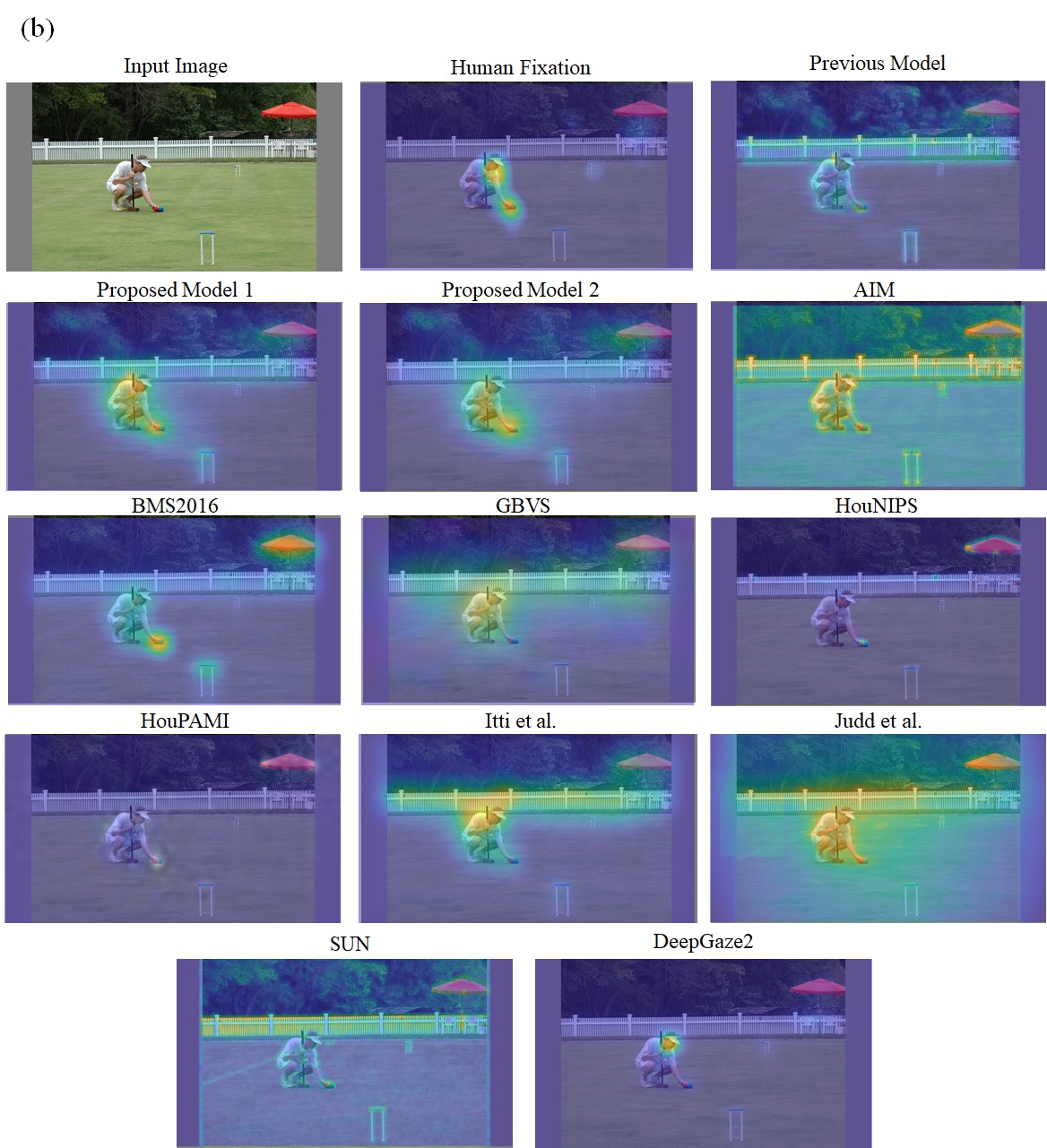


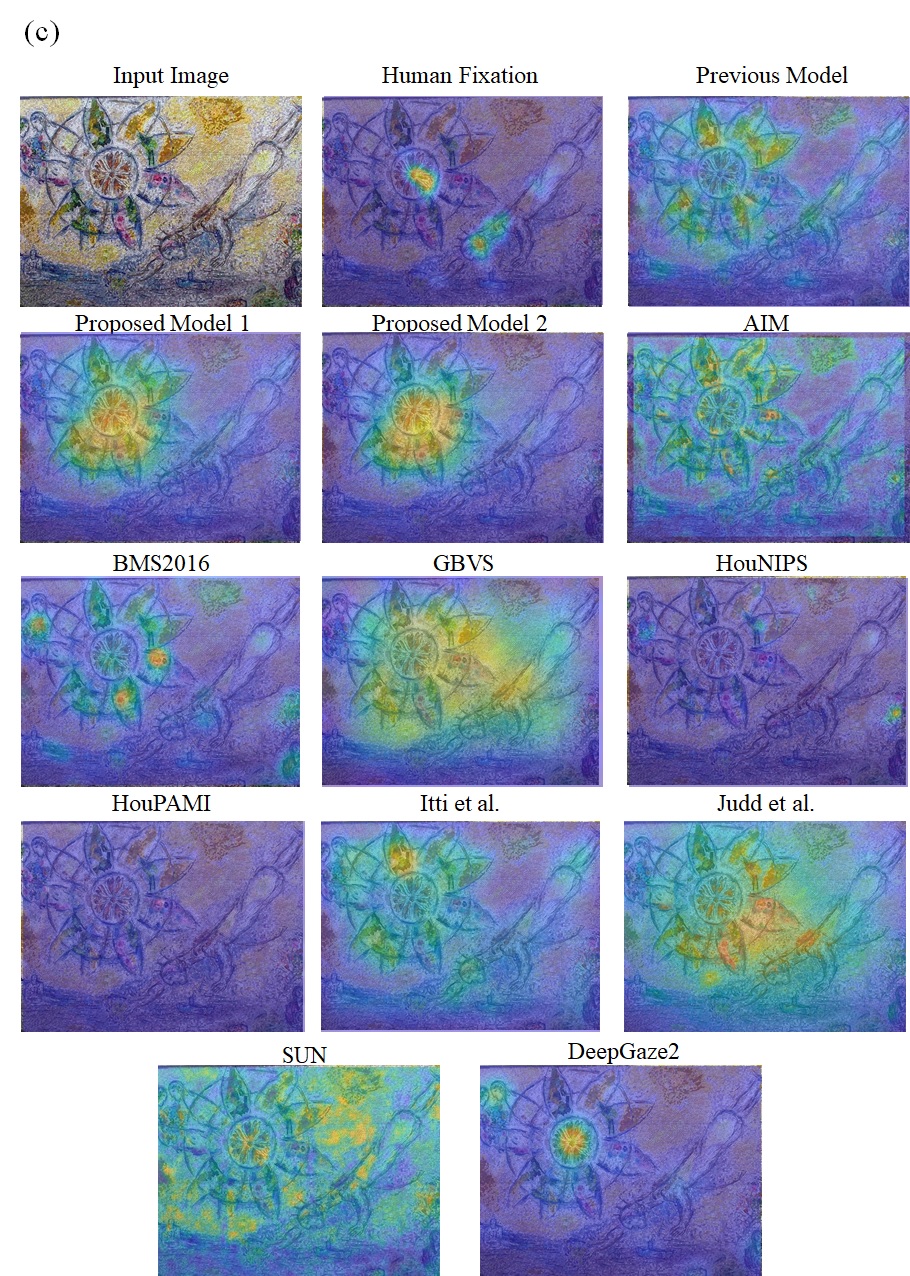


Supplementary Figure 2. Non-blurred saliency maps.

**Reference**

Adelson, E. H., & Bergen, J. R. (1985). Spatiotemporal energy models for the perception of motion. *Journal of the Optical Society of America A*, *2*(2), 284. https://doi.org/10.1364/JOSAA.2.000284

Borji, A., Sihite, D. N., & Itti, L. (2013). Quantitative Analysis of Human: Model Agreement in Visual Saliency Modeling-A Comparative Study. *IEEE Transactions on Image Processing*, *22*(1), 55–69. https://doi.org/10.1109/TIP.2012.2210727

Heeger, D. J. (1992). Normalization of cell responses in cat striate cortex. *Visual Neuroscience*, *9*(2), 181–197. https://doi.org/DOI: 10.1017/S0952523800009640

Heeger, D. J. (1993). Modeling simple-cell direction selectivity with normalized, half-squared, linear operators. *Journal of Neurophysiology*, *70*(5), 1885–1898.

Itti, L., Koch, C., & Niebur, E. (1998). A model of saliency-based visual attention for rapid scene analysis. *IEEE Transactions on Pattern Analysis & Machine Intelligence*, *11*, 1254–1259.

Morrone, M. C., & Burr, D. C. (1988). Feature detection in human vision: a phase-dependent energy model. *Proceedings of the Royal Society of London. Series B, Containing Papers of a Biological Character. Royal Society (Great Britain)*, *235*(1280), 221–245. https://doi.org/10.1098/rspb.1988.0073

Russell, A. F., Mihalaş, S., von der Heydt, R., Niebur, E., & Etienne-Cummings, R. (2014). A model of proto-object based saliency. *Vision Research*, *94*, 1–15. https://doi.org/10.1016/j.visres.2013.10.005

Zhang, L., Tong, M. H., Marks, T. K., Shan, H., & Cottrell, G. W. (2008). SUN: A Bayesian framework for saliency using natural statistics. *Journal of Vision*, *8*(7), 32. http://dx.doi.org/10.1167/8.7.32

Zhou, H., Friedman, H. S., & von der Heydt, R. (2000). Coding of border ownership in monkey visual cortex. *The Journal of Neuroscience*, *20*(17), 6594–6611. https://doi.org/10.1523/JNEUROSCI.2797-12.2013
